# Prenatal Exposure to Chemical Mixtures and Inhibition among Adolescents

**DOI:** 10.3390/toxics9110311

**Published:** 2021-11-16

**Authors:** Anna V. Oppenheimer, David C. Bellinger, Brent A. Coull, Marc G. Weisskopf, Michele Zemplenyi, Susan A. Korrick

**Affiliations:** 1 Department of Environmental Health, Harvard T.H. Chan School of Public Health, Boston, MA 02115, USA; david.bellinger@childrens.harvard.edu (D.C.B.); bcoull@hsph.harvard.edu (B.A.C.); mweissko@hsph.harvard.edu (M.G.W.); susan.korrick@channing.harvard.edu (S.A.K.); 2 Department of Psychiatry, Boston Children’s Hospital, Boston, MA 02115, USA; 3 Department of Biostatistics, Harvard T.H. Chan School of Public Health, Boston, MA 02115, USA; mzemplenyi@gmail.com; 4 Department of Epidemiology, Harvard T.H. Chan School of Public Health, Boston, MA 02115, USA; 5 Channing Division of Network Medicine, Brigham and Women’s Hospital, Harvard Medical School, Boston, MA 02115, USA

**Keywords:** prenatal exposures, chemical mixtures, organochlorines, metals, inhibition, executive function, adolescent neurodevelopment

## Abstract

Inhibition, one of the building blocks of executive function, is the ability to focus one’s attention despite interference from external stimuli. It undergoes substantial development during adolescence and may be susceptible to adverse impacts of prenatal exposure to chemical mixtures, yet few studies have explored this association. The New Bedford Cohort (NBC) is a birth cohort of residents living near the New Bedford Harbor Superfund site in Massachusetts. Among adolescents from the NBC, we investigated the association of biomarkers of prenatal exposure to organochlorines (DDE, HCB, PCBs) and metals (Pb, Mn) with inhibition, assessed with the Delis–Kaplan Executive Function System Design Fluency (non-verbal task) and Color–Word Interference (verbal task) subtests. An exploratory mixtures analysis using Bayesian kernel machine regression (BKMR) informed a traditional multivariable regression approach. NBC adolescents are diverse with 29% non-white and 31% in a low-income household at birth. Cord serum organochlorine concentrations and cord blood metals concentrations were generally similar to other birth cohorts. In BKMR models, we observed a suggestive adverse association of the chemical mixture with Color–Word Interference but not Design Fluency. In covariate-adjusted linear regression models including all five chemical exposure measures, a doubling of cord blood Mn was associated with poorer Color–Word Interference completion time scaled scores (difference = −0.74; 95% CI: −1.34, −0.14). This study provided evidence of an adverse joint association between prenatal exposure to a five-chemical mixture and verbal inhibition in adolescence with exposure to Mn potentially driving this overall association.

## 1. Introduction

Executive functions are mental processes that form the basis of higher-level cognition, including problem-solving, planning, and reasoning [1]. The three core executive functions are inhibition, working memory, and cognitive flexibility [2]. Inhibition, the focus of the present study, is the ability to resist impulse and to focus one’s attention, behavior, and thoughts, despite external stimuli [1]. Although some executive function development begins at a young age, inhibition undergoes substantial evolution during adolescence. This evolution parallels structural and functional changes in the pre-frontal cortex, a part of the brain critical for most executive functions that occur in this age group [3]. Poor inhibition skills in childhood predict physical health problems and substance dependence, as well as lower socioeconomic position and income earned in adulthood [4]. In addition, altered inhibition is associated with a number of mental health disorders including anxiety, depression, and panic disorder [5]. Therefore, identifying modifiable risk factors associated with poorer inhibition skills may diminish the impact of such disorders.

Epidemiologic studies have provided evidence that prenatal exposures to environmental contaminants may be associated with cognitive impacts throughout the life course. The fetus is not well-protected from some environmental exposures as the placenta does not block the maternal transmission of pregnancy exposure to many environmental toxicants including organochlorines and some metals, which are ubiquitous in the environment [6,7,8,9]. In utero, the developing brain undergoes rapid neurological growth and is, therefore, highly sensitive to potential injury from toxic chemicals that may result in long-term neurotoxic impacts.

Several studies have analyzed associations between prenatal environmental chemical exposures and inhibition. Prenatal exposures to organochlorines such as dichlorodiphenyldichloroethylene (DDE), hexachlorobenzene (HCB), and polychlorinated biphenyls (PCBs) have not been studied in relation to inhibition among adolescents specifically. However, among younger children in the Great Lakes region of the United States, two prospective cohort studies found evidence of an association between cord serum PCB levels and poor inhibition measured by psychometric tests of impulse control such as errors of commission on a Continuous Performance Test (CPT) and perseverative errors on the Wisconsin Card Sorting Test (WCST) [10,11,12]. In contrast, in the New Bedford Cohort (NBC), researchers did not find errors of commission on the Neurobehavioral Examination System 2 (NES2)-CPT to be adversely associated with cord serum DDE or PCB concentrations among 8-year-olds who had lower or similar exposure levels, respectively, to the two Great Lakes cohorts [13,14]. Of note, the NES2-CPT is less sensitive to errors of commission than some other CPT instruments [15]. Prenatal exposure to metals may also adversely impact childhood inhibition skills. Manganese (Mn) is an essential trace element necessary for proper brain functioning, though it can be neurotoxic at high levels [16]. The impact of prenatal exposure to Mn on inhibition has not been well-studied among adolescents, but in an exploratory study of younger children, deciduous tooth Mn levels were associated with multiple measures of behavioral disinhibition assessed with a forbidden toy task, a CPT, and a children’s Stroop Test at ages 36 and 54 months [17]. Finally, in a study of a high fish-eating population in the Seychelles, pre- and post-natal exposures to methylmercury (MeHg) measured in maternal and participant hair, respectively, were not found to be associated with inhibition, as measured by the Stroop Color–Word Test among 24-year-olds [18].

Exposure to chemical contaminants rarely occurs independently [19] and co-exposure to chemical mixtures may result in different, often worse, health effects than single chemical effects [20]. Only one study has assessed the association of a chemical mixture with inhibition. In a prospective cohort of children from Arctic Quebec, researchers found that the adverse association between current blood Pb and a child’s ability to inhibit a response in a Go/No-Go task was stronger in children with lower current MeHg and PCB exposures [21]. Other studies have assessed the relation of metal mixtures or metal-PCB mixtures with general executive function or other specific components of executive function, such as working memory. In a prospective cohort study based in Spain, prenatal co-exposure to a metal mixture (composed of cobalt, copper, As, cadmium, antimony, thallium, and Pb) measured in maternal urine during pregnancy was not associated with McCarthy Scales of Children’s Abilities (MSCA) executive function scores among 4-year-olds [22]. In a cross-sectional study of 8 to 11-year-old children from Bangladesh, researchers found that blood Mn was associated with lower Wechsler Intelligence Scale for Children (WISC-IV) working memory scores, but they did not observe a significant interaction between Mn and As [23]. Finally, in a birth cohort study based in the Faroe Islands, among participants with low cord blood levels of MeHg, high cord blood Pb concentrations were associated with lower Digit Span Backward scores, a measure of working memory, at age 14 [24].

In summary, many studies have linked prenatal exposures to organochlorines and metals with decrements in executive function among children. Few have focused on inhibition or on adolescence, when the impact of earlier exposures on executive function may become most readily apparent due to it being a time of substantial executive function development. In addition, even though it has been well-established that the developing brain may be exposed to multiple pollutants simultaneously in utero, few studies have assessed the impact of prenatal exposure to mixtures of prevalent neurotoxic chemicals on executive function and only one has focused on inhibition. Therefore, the purpose of this study was to address this key gap in the literature by investigating the association of prenatal exposure to a prevalent chemical mixture of organochlorines (DDE, HCB, PCBs) and metals (Pb, Mn, MeHg, As) with detailed measures of inhibition among adolescents.

## 2. Materials and Methods

### 2.1. Study Population

The New Bedford Cohort (NBC) is an ongoing, prospective birth cohort study originally designed to assess the effects of prenatal exposures to common chemical pollutants on child development. Between 1993 and 1998, 788 mother-infant pairs were recruited and enrolled in the study shortly after birth at St. Luke’s Hospital, New Bedford, Massachusetts. Mothers were eligible to participate if they were at least 18 years old, spoke English or Portuguese, and were living in one of the four towns surrounding the New Bedford Harbor for at least the duration of their pregnancy. This region of southeastern Massachusetts was chosen for study because the New Bedford Harbor, an EPA Superfund site, was highly contaminated with PCBs and metals from local industrial emissions and there was concern about potential chemical exposure risk to surrounding communities [25,26]. Participation exclusion criteria included birth by cesarean section and infants requiring high-grade neonatal care or who were too ill to undergo study neonatal examinations. Biomarkers of prenatal chemical exposure were collected at birth or two weeks later at a postpartum home visit. The NBC study participants have undergone neuropsychological testing periodically since birth. This analysis focuses on the 528 adolescents who participated in 15-year follow-up exams (median age 15.5, range 13.9–17.9 years) between 2008 and 2014, which included psychometric tests of executive function. Of the 528 participants, 373 had complete data on all executive function outcomes and covariates of interest as well as biomarkers of prenatal exposure to DDE, HCB, PCBs, Pb, and Mn. This group will be referred to as Set 1. A subset of 235 participants had complete data on the same measures as those in Set 1, as well as biomarkers of prenatal exposure to MeHg and As, and will be referred to as Set 2.

### 2.2. Chemical Exposure Assessment

Cord blood samples were collected at birth, centrifuged, and the serum fraction removed and stored at −20 degrees Celsius prior to analyses at the Harvard T.H. Chan School of Public Health Organic Chemistry Laboratory (Boston, MA, USA). After liquid-liquid extraction, cord serum was analyzed for DDE, HCB, and 51 individual PCB congeners using gas chromatography with electron capture detection [25,27,28]. For this analysis, we used the sum of the four most prevalent PCB congeners—118, 138, 153, 180 (ΣPCB_4_)—as they were measured with the least measurement error and are most frequently used to assess congener-specific health effects in other population-based studies [28]. For the organochlorines, the limits of detection (LODs) ranged from 0.001 ng/g to 0.07 ng/g serum. Organochlorine chemical analyses were highly reproducible with within-batch coefficients of variation ranging from 5% to 7.5% and the between-batch coefficients of variation ranging from 20% to 39% over 5 years of analysis [27].

Cord whole blood samples were also collected at birth and refrigerated prior to metals analyses at the Harvard T.H. Chan School of Public Health Trace Metals Laboratory (Boston, MA, USA). Blood Pb and Mn were measured using isotope dilution (ID) inductively coupled plasma mass spectrometry (ICP-MS, Sciex Elan 5000, Perkin Elmer, Norwalk, CT, USA) and external calibration on a dynamic reaction cell-inductively coupled plasma-mass spectrometer (DRC-ICP-MS, Elan 6100, Perkin Elmer, Norwalk, CT, USA), respectively. Concentrations were reported as the mean of five replicate measurements. Procedural blanks, duplicates, spiked samples, standard reference material (NIST SRM 955b Pb in blood; NIST SRM 1643d trace elements in water) and biological reference material (ICP03B-05 and ICP03B-02 multi-elements in human blood from INSPQ/Laboratoire de Toxicologie, Quebec, Canada were used for quality control (QC) monitoring. Recovery rates for QC and spiked samples were 90–110% and precision > 95%. The LOD was 0.02 µg/dL.

Maternal hair samples were cut from the occiput, on average, two weeks postpartum and analyzed for mercury (Hg) at the Harvard T.H. Chan School of Public Health Trace Metals Analysis Laboratory. Prior to analysis, hair samples were cleaned using sonication, rinsed with distilled deionized water, and dried for 24 h [29]. Where the proximal end was identified, the three centimeters closest to the scalp, which approximates Hg exposure in the third trimester of pregnancy, were analyzed for total Hg by atomic absorption spectroscopy using a DMA-80 Direct Mercury Analyzer (Milestone Inc., Monroe, CT, USA). Hair total Hg concentrations are a reasonable proxy for hair MeHg levels [30]. Quality control procedures included daily calibration verification, procedural blanks, and certified reference material (GBW 09101 human hair, Shanghai Institute of Nuclear Research Academia Sinica, Shanghai, China) [31]. Recovery rates for quality control standards were 90–110%, precision > 95% and the average LOD was 50 ng/g of hair [29].

Arsenic (As) was measured in maternal toenails collected, on average, two weeks after birth, to approximate As exposure throughout pregnancy. Analyses were performed at the Trace Element Analysis Laboratory at Dartmouth College (Hanover, NH, USA). The toenail samples were cleaned by sonication, rinsed with distilled deionized water, and dried prior to analysis. The samples were then weighed and digested with 1 mL of HNO_3_ acid for 24 h at room temperature. Analyses were performed using an external calibration method on a dynamic reaction cell-inductively coupled plasma-mass spectrometer (Agilent 7700x ICP-MS, Santa Clara, CA, USA), which used 5 standards at concentrations ranging from 0 to 50 ng/mL. Quality control procedures included analyses of daily calibration verification, a procedural blank, and certified reference material. Coefficients of variation for reference standards were less than 15% for toenail As [32]. The average LOD for As in toenails was 0.03 ng/g.

### 2.3. Inhibition Assessment

At the NBC 15-year follow-up, a trained study examiner administered six subtests of the Delis–Kaplan Executive Function System (D-KEFS) [33]. Inhibition was assessed using two of these subtests: Design Fluency: Empty Dots Only condition (a non-verbal task) and Color–Word Interference: Inhibition condition (a verbal task). In Design Fluency: Empty Dots Only, the examinee is presented with response boxes that contain 5 filled dots and 5 empty dots and the examinee must inhibit connecting filled dots and only connect those dots that are empty to create as many different designs as possible within 60 s. Performance was measured with the total correct scaled score in the main analysis and the total errors raw score in a secondary analysis. Total errors were a sum of total set loss designs (creating designs that did not follow the rules of the task) and total repetition errors (creating the same design more than once). For Design Fluency, higher total correct scaled scores and lower total error raw scores mean better performance. In Color–Word Interference: Inhibition, the examinee must inhibit reading words denoting colors to name dissonant ink colors in which those words are printed. For example, if the word ‘red’ is printed in green ink, the examinee must inhibit reading the word ‘red’, the prepotent response, and, instead, say the word ‘green’. Performance was measured with the total completion time scaled score in the main analysis. In secondary analyses, performance was also measured with: (1) the total errors raw score; and (2) a score that combined total completion time raw scores and total errors raw scores to simultaneously capture both speed and accuracy of performance. Integrating these two scoring criteria allowed us to create a more comprehensive representation of Color-Word Interference performance than is possible when considering each scoring criterion separately, as is commonly done. For the analysis, this combined score was dichotomized wherein the best performance group included those who had performed better than the population median score for both dimensions (total completion time raw score < 52.0 s and total errors raw score < 2), while the poor performance group included the remaining participants. For Color–Word Interference, higher completion time scaled scores, lower completion time raw scores, and lower total error raw scores mean better performance.

### 2.4. Covariate Assessment

Periodic medical record review as well as parental and child self-reported questionnaire data were used to obtain and update demographic, health, and lifestyle information for the study participants. At birth, a trained study nurse reviewed medical records to obtain infant race/ethnicity, birth weight, gestational age, information about the mother’s pregnancy and delivery, and the baby’s initial pediatric examination and any laboratory test results after delivery [29]. Approximately two weeks later, participating mothers were interviewed at a home visit to gather information about maternal pregnancy diet, smoking, alcohol, and drug use, medical and reproductive histories, infant feeding, demographic information, income, and occupational and educational histories for both parents. Medical record reviews and questionnaire data were updated at 8-year and 15-year follow-up assessments. These follow-up assessments also included a home visit to assess the quality of the child’s home environment and parent-child relationship using the Home Observation for Measurement of the Environment (HOME) assessment instrument questionnaire [34]. Maternal IQ was assessed using the Kaufman Brief Intelligence Test (KBIT) [35] either at the 8-year or 15-year follow-up.

We also constructed a prenatal social disadvantage index (PNSDI) composed of the sum of five adverse social or economic exposures at the time of the child’s birth: mother unmarried, mother’s education as high school graduate or less, father’s education as high school graduate or less, annual household income less than USD 20,000, and mother’s age at birth less than 20 years.

### 2.5. Statistical Analysis

The main exposure of interest was a chemical mixture composed of biomarkers of prenatal exposure to DDE, HCB, ΣPCB_4_, Pb and Mn. In secondary analyses, we added biomarkers of prenatal exposure to MeHg and As to the mixture. MeHg and As were not included in the primary analyses in order to improve power, as MeHg and As concentrations were measured in maternal hair and nails collected two weeks postpartum rather than at delivery, which resulted in some missingness.

Regression diagnostics supported log-transforming chemical exposures to reduce the influence of extreme values. Log_2_-transformation was used so all effect estimates represent a two-fold increase in exposure levels. As an exploratory tool, we first used Bayesian kernel machine regression (BKMR) to assess potential non-linear dose-response relationships and interactions among exposures in determining inhibition skills. BKMR is an exposure–response surface estimation technique for mixtures that models the relationship between a high-dimensional set of predictors and an outcome using a flexible exposure–response function [36]. Using Markov chain Monte Carlo (MCMC) for Bayesian inference, this method can overcome issues such as collinearity and overfitting that can be problematic with other approaches to exposure mixture assessments [36]. Due to the high dimensionality of the exposure mixture, it is not possible to visualize the entire exposure–response function resulting from a BKMR analysis. However, it is possible to visualize the relationship between each individual exposure and an outcome or the joint effect of two exposures on an outcome, while fixing the other exposures to pre-specified values, such as the median of each distribution. The resulting visualizations of the exposure–response relationship facilitate identification of non-linear exposure–outcome associations and potential interactions among exposures.

Specifically, we visually inspected plots of the estimated exposure–response functions and 95% credible intervals of the five main exposures (DDE, HCB, ΣPCB_4_, Pb and Mn) and the seven secondary exposures (5 main exposures plus MeHg and As) with inhibition performance while assigning the remaining exposures to their median value. Where the exposure–response functions appeared non-linear, we included a quadratic term for the chemical in linear regression models that included the main effect of all the exposures, covariates, and a quadratic term and tested for the quadratic effect using a likelihood ratio test with 2 degrees of freedom. Next, we visually inspected plots of the estimated exposure–response functions between one of the five main exposures or seven secondary exposures and inhibition performance, where a second exposure was fixed at varying levels of exposure while the remaining exposures were assigned to their median value. If the slope of each chemical was similar at varying levels of the second exposure, we interpreted this pattern as indicating the absence of an interaction between the two chemicals. However, if the slope of a chemical differed at varying levels of a second exposure, we fit covariate-adjusted linear regression models with and without an interaction term and compared model fit using a likelihood ratio test. BKMR analyses were also used to assess the joint association of the chemical mixture with each of the inhibition subtests. All analyses were conducted using R version 3.6.0 [37], with BKMR analyses conducted using the *bkmr* package in R [38].

We used the results of our BKMR analyses to inform parametric linear regression models estimating the association of the five or seven exposures with the inhibition outcomes, while adjusting for covariates. All five (Set 1) or seven (Set 2) exposures were included in the models simultaneously. In the main (Set 1) analyses, we subsequently included chemical-sex or chemical-PNSDI interaction terms in the linear regression models then stratified by sex and PNSDI. We then analyzed the association between the five-chemical mixture and Design Fluency total errors raw score, Color–Word Interference total errors raw scores, and the Color–Word Interference overall performance measure. Specifically, as the distributions of Design Fluency and Color–Word Interference total error raw scores were consistent with over-dispersed count data, negative binomial regression was used to estimate the relationship of these outcomes with chemical mixtures using rate ratios (RRs). Meanwhile, as the Color–Word Interference overall performance outcome was binary, we used logistic regression to assess the odds of being in the poor compared to the best performance group. Once again, all five (Set 1) exposures were included in the models simultaneously.

To account for potential selection bias due to loss to follow-up, we used linear regression with inverse probability weights (IPW) for censoring [39]. IPW is a technique in which individuals in the analytic group are weighted based on the inverse of the probability of being included in the analysis, given their particular exposure and covariate values. The following exposures and covariates were chosen for the IPW missingness model based on their prediction of loss to follow-up for this analysis as well as for other longitudinal cohort studies reported in the literature: biomarker levels of DDE, HCB, ΣPCB_4_, and Pb and socio-demographic characteristics of the mother at birth such as education and household income and child characteristics such as race/ethnicity and sex. This weighting procedure created a pseudo-population that represented the original source population that was recruited to the NBC at birth but, by definition, did not include those who were missing covariates used to create the weights. The distributions of non-missing covariates were comparable between the source population with complete data (*n* = 622) and the original cohort (*n* = 788) supporting the representativeness of our population weights. We used stabilized IPW, trimmed at the 2.5th and 97.5th percentile [39].

**Figure 1:**
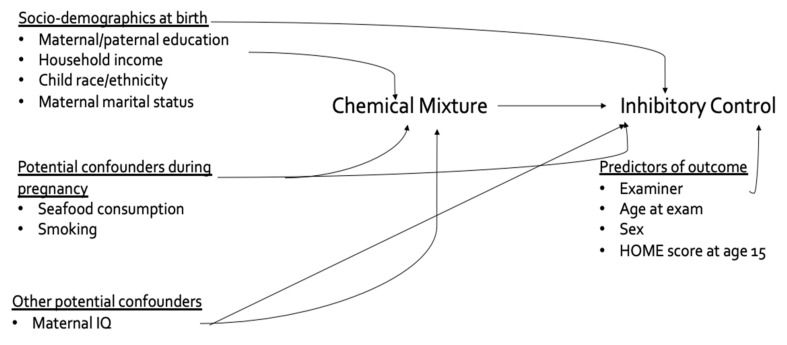
Directed acyclic graph (DAG) describing potential confounders and predictors of inhibition that were included in adjusted models. The underlined portion describes the different categories of covariates included in this model.

Potential covariates were selected using a directed acyclic graph (DAG) (Figure 1) that was developed based on a review of the literature regarding potential confounders of the relationship of prenatal organochlorine and metal exposures with cognition. We also considered covariates that had been previously found to predict cognitive outcomes in the NBC. Based on DAGs and priors, the following covariates were included in the final models: adolescent race/ethnicity, sex, age at exam and HOME score; maternal marital status at birth, IQ, seafood consumption and smoking during pregnancy; maternal and paternal education and household income at child’s birth; and examiner. Characteristics of participants who were included in the main and secondary analyses were compared to those not included using t-tests, Wilcoxon rank sum tests, and Chi-square tests where appropriate.

## 3. Results

### 3.1. Study Population Characteristics

Table 1 describes the characteristics of adolescents in the main analysis group who had complete executive function outcome measures, covariates, and biomarkers of exposure (Set 1: DDE, HCB, ΣPCB_4_, Pb, Mn) and those who were excluded from the main analysis.

Table S1 describes the characteristics of adolescents in the secondary analysis group who had complete executive function outcome measures, covariates, and biomarkers of exposure (Set 2: DDE, HCB, ΣPCB_4_, Pb, Mn, MeHg, As) and those who were excluded from the secondary analysis. The NBC population included in the main analysis was socio-demographically diverse with 29.5% of participants being non-white, 50.9% having mothers with less than or equal to a high school education at the time of their birth, and 30.8% having an annual household income of less than USD 20,000 per year at the time of their birth (Table 1). Those included in both the main and secondary analyses had, on average, characteristics consistent with greater sociodemographic advantage compared to those excluded. For example, compared to those excluded, participating adolescents were more likely to live in a household with higher income at the time of their birth, their mothers had higher IQs and were more likely to be married at birth, and both parents had higher educational attainment. In addition, 15-year follow-up participants in this analysis had higher serum levels of DDE and lower cord blood Pb levels than those excluded from the study. Lastly, Set 2 adolescents generally performed better on tests of inhibition than those who were excluded from Set 2 analyses (Table S1).

### 3.2. Chemical Exposure Measures

Biomarker concentrations of organochlorines and metals in the NBC study participants were similar to the general population of the United States and Canada, with the exception of total hair Hg concentrations which were similar to those observed in high fish-eating populations [14,40,41,42,43]. In Set 1, the organochlorines were moderately correlated with each other (Spearman r: 0.4–0.6), Pb was weakly correlated with the organochlorines and Mn (Spearman r: 0.1–0.2), and Mn was not correlated with the organochlorines. In Set 2, MeHg was moderately correlated with the organochlorines (Spearman: 0.2–0.5) and weakly correlated with Pb (Spearman r = 0.1) but not with the other metals. As was not correlated with the organochlorines or other metals.

### 3.3. Inhibition Measures

As expected, given that higher scaled scores mean better performance, while higher error raw scores mean worse performance, Design Fluency total correct scaled scores and total error raw scores were weakly negatively correlated (Spearman r = −0.04), while Color–Word Interference total completion time scaled scores and total errors raw scores were moderately negatively correlated (Spearman r = −0.4). Across the two inhibition subtests, Design Fluency total correct scaled scores and Color–Word Interference completion time scaled scores were weakly positively correlated (Spearman r = 0.2).

### 3.4. BKMR Analyses of Prenatal Exposure to Five- and Seven-Chemical Mixtures with Inhibition

**Figure 2:**
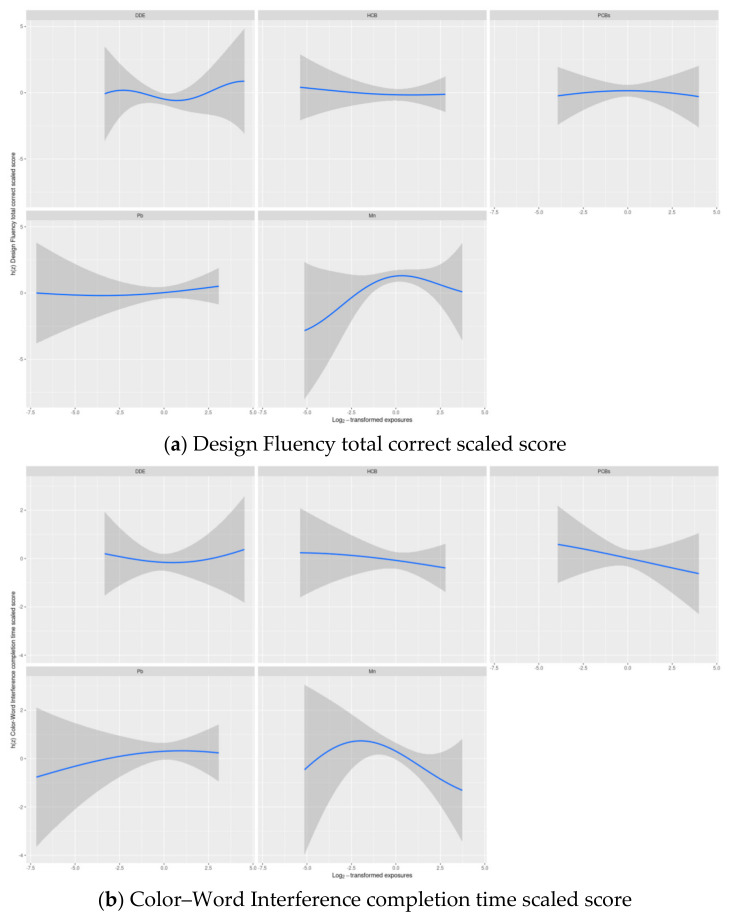
Estimated exposure-response functions and 95% credible intervals ^1^ of each of the five main exposures in Set 1^2^ with the Delis–Kaplan Executive Function System inhibition scaled scores: (**a**) Design Fluency total correct scaled score and (**b**) Color–Word Interference completion time scaled score, where all remaining exposures are assigned to their median value among adolescents in the main analysis group. ^1^ Exposures have been log2-transformed and models have been adjusted for child race, sex, age at exam, year of birth and HOME score; maternal marital status at child’s birth, IQ, seafood consumption during pregnancy, and smoking during pregnancy; maternal and paternal education and annual household income at child’s birth; and study examiner. ^2^ Set 1: complete outcome, covariate and exposure data for DDE, HCB, ΣPCB_4_, Pb and Mn, *n* = 373. Abbreviations: DDE: dichlorodiphenyldichloroethylene; HCB: hexachlorobenzene; ΣPCB_4_: Sum of 4 PCB congeners (118, 138, 153, 180); Pb: lead; Mn: manganese.

Visual inspection of BKMR results suggested potential non-linear associations of DDE and Mn with Design Fluency scaled scores and Mn with Color–Word Interference scaled scores (Figure 2, Figure S1).

**Figure 3:**
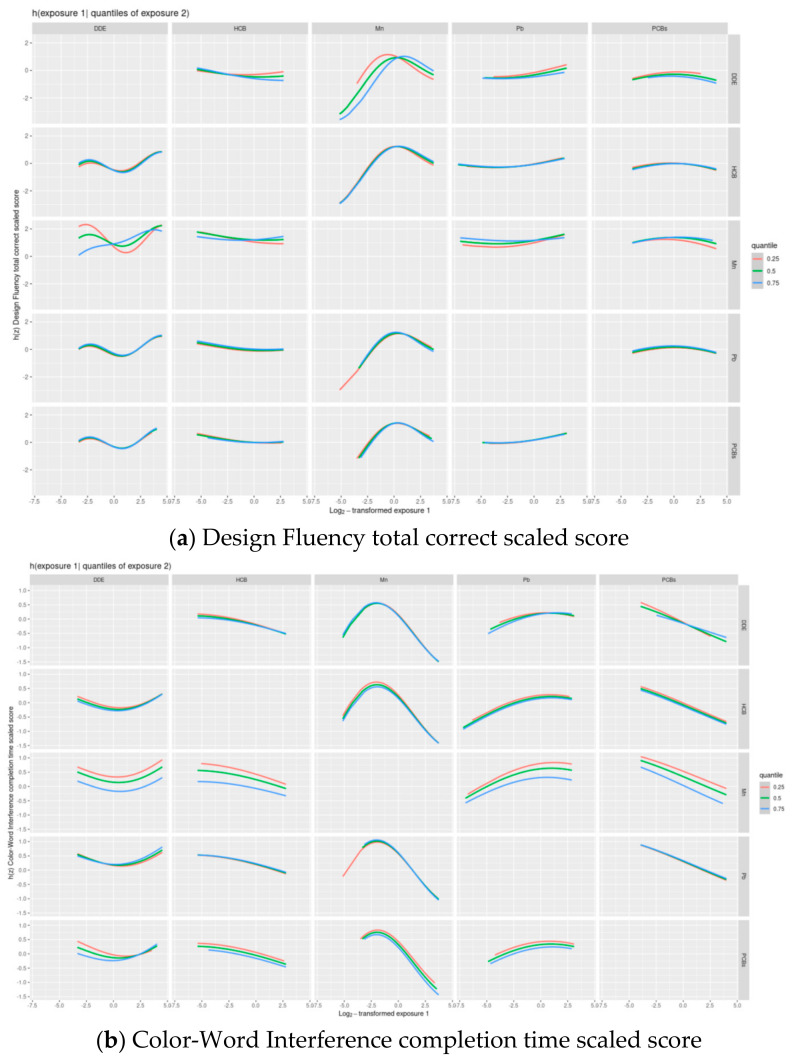
Exposure-response functions ^1^ associating each of the five main exposures (Set 1^2^) and a second exposure fixed at various quantiles with the Delis–Kaplan Executive Function System inhibition scaled scores: (**a**) Design Fluency total correct scaled scores and (**b**) Color–Word Interference completion time scaled score, while the remaining exposures are assigned to their median value among adolescents in the main analysis group. ^1^ Exposures have been log2-transformed and models have been adjusted for child race, sex, age at exam, year of birth, and HOME score; maternal marital status at child’s birth, IQ, seafood consumption during pregnancy, and smoking during pregnancy; maternal and paternal education and annual household income at child’s birth; and study examiner. ^2^ Set 1: complete outcome, covariate and exposure data for DDE, HCB, ΣPCB_4_, Pb and Mn, *n* = 373.Abbreviations: DDE: dichlorodiphenyldichloroethylene; HCB: hexachlorobenzene; ΣPCB_4_: Sum of 4 PCB congeners (118, 138, 153, 180); Pb: lead; Mn: manganese.

Wald tests only supported including a quadratic term for Mn in the Design Fluency model, therefore in the subsequent main analyses, a quadratic term for Mn was included in the model for Design Fluency. The BKMR results also provided evidence of an interaction between DDE and Mn in their association with Design Fluency, which was confirmed with a likelihood ratio test (Figure 3, Figure S2).

**Figure 4:**
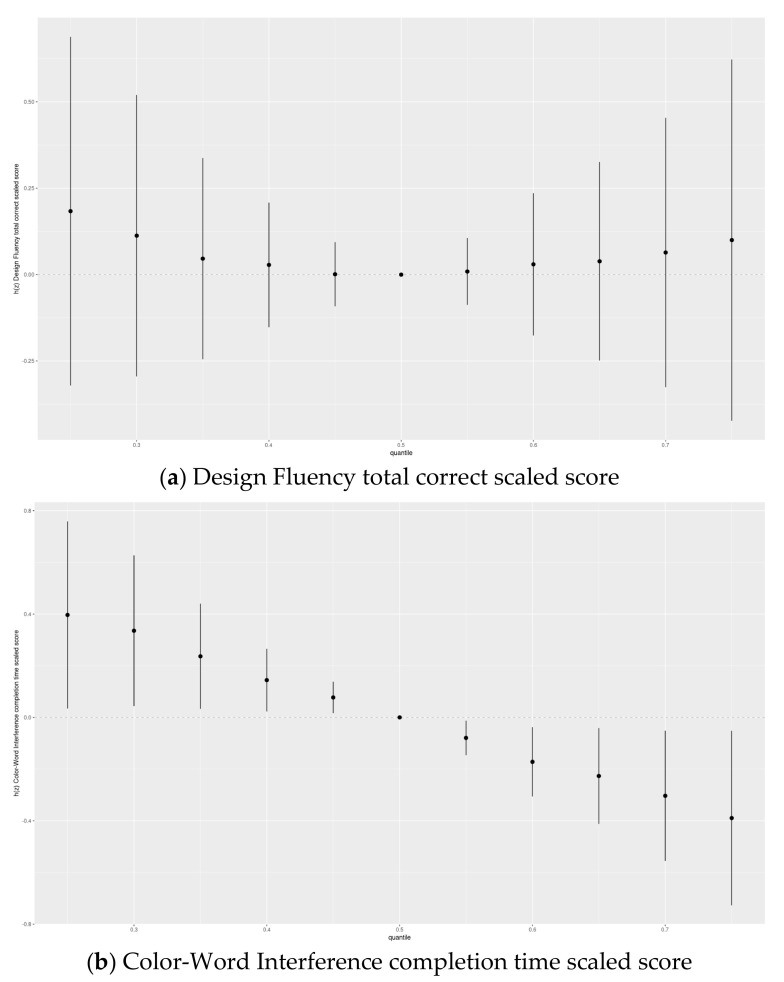
Joint association (estimates and 95% credible intervals) ^1^ of the five-chemical mixture ^2^ (DDE, HCB, ΣPCB_4_, Pb and Mn) with the Delis–Kaplan Executive Function System inhibition scaled scores: (**a**) Design Fluency total correct scaled score and (**b**) Color–Word Interference completion time scaled scores among adolescents in the main analysis group. Chemical mixture levels at each percentile are compared to a mixture with each component at its median level. ^1^ Exposures have been log2-transformed and models have been adjusted for child race, sex, age at exam, year of birth, and HOME score; maternal marital status at child’s birth, IQ, seafood consumption during pregnancy, and smoking during pregnancy; maternal and paternal education and annual household income at child’s birth; and study examiner. ^2^ Set 1: complete outcome, covariate and exposure data for DDE, HCB, ΣPCB_4,_ Pb and Mn, *n* = 373. Abbreviations: DDE: dichlorodiphenyldichloroethylene; HCB: hexachlorobenzene; ΣPCB_4_: Sum of 4 PCB congeners (118, 138, 153, 180); Pb: lead; Mn: manganese.

Therefore, the Design Fluency model included:

DDE + Mn + Mn^2^ + DDE ∗ Mn + DDE ∗ Mn^2^

in the linear regression model along with the remaining exposures and covariates. Finally, we used BKMR to assess the joint association of the chemicals with the scaled scores of the inhibition subtests (Figure 4, Figure S3) by visually comparing the effect of the chemical mixture at various percentiles to their median levels.

Visual inspection of BKMR results supported the findings of our parametric multi-exposure models wherein Mn was adversely associated with inhibition but other chemicals in the mixture were not (see Section 3.5). For BKMR assessment of joint impacts for Set 1 exposures, there appeared to be an adverse overall association of the chemical mixture with Color–Word Interference but not Design Fluency. In Set 2 exposures, there was evidence of adverse joint associations of the chemicals with both inhibition subtests.

### 3.5. Linear Regression Analyses of the Association of Prenatal Exposure to Five Chemicals with Inhibition

In the main linear regression analyses (Set 1), we observed that a doubling of cord blood Mn was associated with lower Color–Word Interference completion time scaled scores (difference = −0.74; 95% CI: −1.34, −0.14) (Table 2).

**Figure 5:**
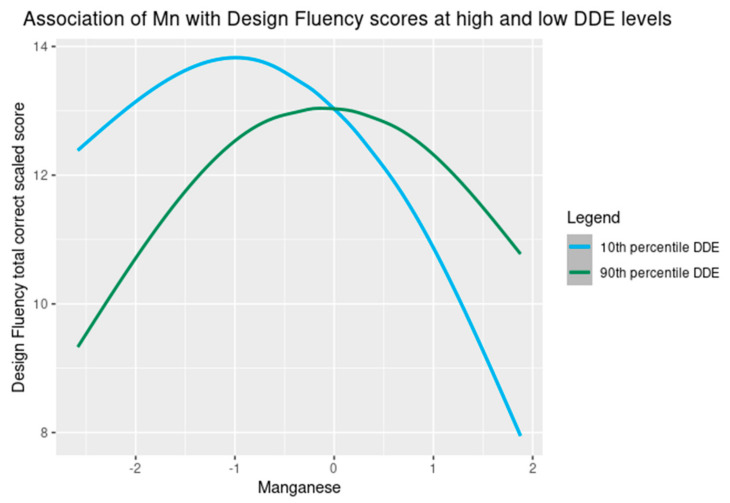
The association of prenatal Mn exposure with Design Fluency total correct scaled scores ^1^ when DDE levels are at the 10th and 90th percentile among adolescents in the main analysis group (Set 1) ^2^. ^1^ Exposures have been log2-transformed and models have been adjusted for DDE, HCB, ΣPCB_4_, Pb, child race, sex, age at exam, year of birth, and HOME score; maternal marital status at child’s birth, IQ, seafood consumption during pregnancy, and smoking during pregnancy; maternal and paternal education and annual household income at child’s birth; and study examiner. ^2^ Set 1: complete outcome, covariate and exposure data for DDE, HCB, ΣPCB_4_, Pb and Mn, *n* = 373. Abbreviations: DDE: dichlorodiphenyldichloroethylene; HCB: hexachlorobenzene; ΣPCB_4_: sum of 4 PCB congeners (118, 138, 153, 180); Pb: lead; Mn: manganese.

To further examine the potential interaction between DDE and Mn, we plotted the association of Mn with Design Fluency total correct scaled scores among those with low (10th percentile) and high (90th percentile) DDE levels (Figure 5).

We found nonlinear associations of Mn with Design Fluency regardless of DDE exposure. However, there appeared to be a stronger adverse association of higher concentration of Mn with Design Fluency among those who had low DDE levels compared to those who had high DDE levels (Figure 5). Among the remaining chemical exposures, estimates were largely null with the exception of PCBs, which trended negative though with wide confidence intervals that included the null (Table 2). Results of linear regression analyses using IPW were similar to complete case analyses (Table S2). In summary, in multi-exposure models, only Mn was consistently associated with poorer inhibition as measured by Color–Word Interference and Design Fluency. In the latter case, adverse associations were non-linear (observed at higher Mn levels) and most evident in the setting of low DDE exposures.

### 3.6. Sex-Stratified Linear Regression Analyses of the Association of Prenatal Exposure to Five Chemicals with Inhibition

We did not find any statistically significant chemical-sex interactions in the main linear regression models, although sex-stratified analyses were likely underpowered and therefore did not detect statistically significant sex-specific adverse associations (Table 3).

### 3.7. Social Disadvtange-Stratified Linear Regression Analyses of the Association of Prenatal Exposure to Five Chemicals with Inhibition

We found limited evidence of interaction between the PNSDI and our exposures of interest (Table 4).

The associations of the organochlorine pesticides with inhibition were more adverse among those with a PNSDI ≥ 3 than those with PNSDI < 3, though the reverse was true for PCB-inhibition associations. Any negative associations observed between the metals and inhibition outcomes (e.g., Pb-Design Fluency, Mn-Color–Word Interference) tended to be stronger among those with PNSDI < 3. The sex-stratified and PNSDI-stratified IPW results were similar to the complete case results (Tables S3 and S4). In summary, our findings suggest that exposure to social disadvantage may modify associations of prenatal exposure to organochlorines and metals with inhibition.

### 3.8. Negative Binomial Regression Analyses of the Association of Prenatal Exposure to Five Chemicals with Errors on Inhibition Tasks

Next, we analyzed the association of the five-chemical mixture with Design Fluency and Color–Word Interference total error raw scores, as well as an overall measure of Color–Word Interference that incorporated both speed and accuracy (Table 5 and Table 6). The associations of the chemicals and error scores were largely null, with the exception of an unexpected association between Mn and fewer Design Fluency total errors (Table 5).

### 3.9. Logistic Regression Analyses of the Association of Prenatal Exposure to Five Chemicals with Overall Performance on Color–Word Inhibition Task

We also found suggestive evidence of increased odds of poor Color–Word Interference performance per doubling of cord blood Mn (OR = 1.61; 95% CI: 0.98, 2.64) (Table 6).

Associations of overall Color–Word performance with the other chemicals in the mixtures were imprecise and included the null. IPW results were similar to complete case results (Tables S5 and S6).

### 3.10. Secondary Analyses

The results of the secondary analyses (Set 2) were imprecise, though PCBs and Mn were adversely associated with Color–Word Interference completion time scaled scores (PCBs difference = −0.54; 95% CI: −1.02, −0.06; Mn difference = −0.64; 95% CI: −1.38, 0.11) (Table S7). IPW results were similar (Table S8).

## 4. Discussion

Results of our main analyses provided evidence that prenatal exposure to Mn at the levels seen in the NBC may adversely impact inhibition among adolescents even with adjustment for exposure to multiple prevalent neurotoxic metals and organochlorines (Table 2). Specifically, in multi-exposure models, we observed adverse associations of cord blood Mn with the Color–Word Interference measure of inhibition. Results were largely unchanged when we used IPW to account for potential selection bias due to loss to follow-up. Color–Word Interference, a verbal task, appeared to be more sensitive to Mn exposure than Design Fluency, a non-verbal task. This is consistent with other studies that have found associations between increasing Mn concentrations and poorer performance on verbal cognitive tasks [44,45,46]. Prenatal Mn exposure was also adversely associated with a measure of Color–Word Interference overall performance which accounted for both completion time and total errors raw scores (Table 6). Although more imprecise due to a smaller sample size, the adverse impacts of Mn were still evident in our secondary analyses, which included MeHg and As as part of the chemical mixture (Table S7). Prenatal exposure to Mn has also been associated with decrements in inhibition in a small exploratory study (*n* = 27) where increasing tooth Mn concentrations, reflecting exposure in the 20th week of gestation, were associated with behavioral disinhibition as measured by a Forbidden Toy Task, a CPT, and a children’s Stroop Test at 36 and 54 months [17]. Mechanistic studies support the biological plausibility of these associations. For example, Mn targets the brain’s dopaminergic system [47,48], which plays a key role in inhibition skills [49]. Specifically, animal studies have shown associations of neonatal Mn exposure with altered striatal dopamine levels and dopaminergic receptor functions in the prefrontal cortex and other brain regions involved in inhibition [47,48]. Our findings provide evidence that prenatal exposures to relatively low-level Mn may have adverse impacts on inhibition that persist beyond early childhood into adolescence.

The association of Mn with Design Fluency appeared non-linear and was modified by DDE exposure levels. Previous studies have also found non-linear associations of prenatal Mn with cognition among infants, young children, and adolescents [50,51,52], though this is the first study to find evidence of a DDE-Mn interaction. This nonlinear association may be because Mn is an essential element necessary for proper brain functioning with evidence of neurotoxicity at low and high concentrations [53]. Of note, there is no well-established industrial source of Mn exposure located near the NBC study communities; therefore, NBC participants were likely exposed to Mn via multiple sources including diet [54,55].

In addition, we observed adverse associations of cord serum ΣPCB_4_ with both Design Fluency and Color–Word Interference measures of inhibition; however, these associations were imprecise and included the null. Results were unchanged when we used IPW to account for loss to follow-up. Prenatal PCB exposure has previously been implicated as detrimental to inhibition in several studies among younger children [10,11,12,56]. In a prospective cohort study among Lake Michigan fish eaters who had much higher PCB levels than those seen in the NBC [14], increasing prenatal PCB exposure was associated with poorer response inhibition and greater impulsivity as measured by CPT errors of commission and WCST preservative errors among 11-year-olds who had not been breastfed [10]. In the Oswego-based cohort, which had similar PCB biomarker concentrations to the NBC, increasing prenatal PCB exposure was associated with lower inhibition at ages 4.5 and 9.5 years measured using CPT commission errors, as well as a Differential Reinforcement of Low Rates task administered at the older age [11,12,56]. Interestingly, an analysis of prenatal PCB exposure and CPT errors of commission in the NBC at age 8 years did not find an adverse association [13]. This discrepancy may be, in part, due to variation in CPT tests—by design, the CPT used in the NBC was more sensitive to attention skills rather than inhibition [15].

Among the remaining Set 1 exposures (HCB, DDE, Pb), associations with inhibition outcomes were imprecise and largely null. Although we did not find HCB to be associated with the inhibition outcomes, a longitudinal cohort study in Greece previously found that HCB concentrations in maternal serum during the third trimester of pregnancy were associated with lower executive function and working memory scores on the MSCA among 4-year-olds [57]. However, participants in the NBC study had lower concentrations of HCB than participants in the Greek study. In addition, we assessed inhibition and other executive function measures in adolescence rather than early childhood. It is possible that prenatal HCB exposure does not adversely impact inhibition at the exposure levels seen in the NBC, or that HCB-related impacts do not persist through adolescence.

There was no evidence of an association of DDE with inhibition (Table 2), which may be due to residual negative confounding as could occur from co-exposure to beneficial nutrients in dietary sources of DDE exposure [58]. Although we adjusted for maternal seafood consumption during pregnancy, self-reported diet is imprecise and we did not have information on other potential dietary sources of DDE as well as beneficial nutrients, such as fruit and vegetable consumption.

Finally, Pb was not associated with Design Fluency or Color–Word Interference (Table 2). It is possible that we did not see an adverse association of Pb with inhibition due to negative residual confounding by socioeconomic position. For example, in the NBC study, participants with mothers who were older at the time of their birth tended to have higher cord blood Pb concentrations, however, older mothers were also more likely to attain higher education levels and have a higher household income than younger mothers. Although we adjusted for maternal education and household income, these variables may not fully capture sociodemographic and economic confounding. Including maternal age at birth in models did not change Pb-inhibition associations. In addition, previous studies have not found strong evidence of associations between prenatal Pb exposure and executive functions [59,60]. One longitudinal study that measured cord blood Pb at birth and blood Pb at multiple time points thereafter found that executive function outcomes in mid-childhood were adversely impacted by recent Pb exposure, rather than Pb exposure at birth [60]. These findings suggest that exposure timing may play an important role in the association of Pb with inhibition or other executive functions.

We did not find statistically significant differences in chemical exposure–inhibition associations among different sexes, potentially due to limitations in study power. When we stratified the results by sex, we did see some sexual dimorphism in effect estimates (e.g., HCB and Design Fluency, Mn and both inhibition outcomes), though the more susceptible sex varied by outcome and differences were not statistically significant (Table 3). We found a statistically significant difference in HCB-Design Fluency associations between the two PNSDI categories, with a stronger adverse association among those who had more prenatal social disadvantage (Table 4). This was the general pattern observed for associations between the organochlorine pesticides and inhibition outcomes, but not PCBs where stronger adverse associations were observed among those with less social disadvantage (PNSDI < 3). There was no evidence of effect modification by PNSDI in the associations of the metals with inhibition outcomes. Similar patterns were observed in PNSDI-stratified analyses of the same chemical mixture and working memory outcomes in the New Bedford Cohort [61].

In secondary analyses, in which As and MeHg were included in the chemical mixture, we found that increasing prenatal MeHg exposure was associated with lower Design Fluency scaled scores, but higher Color–Word Interference scaled scores (Table S7). Studies of populations with high dietary MeHg exposure in the Faroe Islands and the Seychelles have not found evidence of adverse associations between prenatal exposure to MeHg and executive function [18,62]. The higher Color–Word Interference scaled scores associated with a doubling of MeHg observed in our study may be due to residual confounding by nutritional benefits of fish consumption, an important source of MeHg exposure [58]. Although we accounted for maternal seafood consumption during pregnancy, there is likely measurement error in the use of self-reported fish intake as a proxy for nutritional confounding in this setting. Meanwhile, associations of As exposure with both inhibition subtests were largely null with wide confidence limits bounding the effect estimates (Table S7). As previously noted, there are few studies of prenatal exposure to As and inhibition. However, two cross-sectional studies have found adverse associations of As exposure with working memory, another executive function in which inhibition plays a role, but at much higher levels of As than in our study population [63,64]. Therefore, further research is necessary to characterize the impact of low-level prenatal As exposures on inhibition.

In this study, the main analysis involved traditional linear regression, while BKMR was used as an exploratory tool. The traditional method was used primarily to improve the interpretability of the results and to be able to compare results with other studies. Meanwhile, BKMR allows for nonlinear and nonadditive effects and can account for multiple comparisons as well as consider joint exposure effects [36]. We used BKMR to visualize the joint association between the chemical mixture and the inhibition outcomes. In this study, we found an adverse joint association of the chemical mixture with Color–Word Interference in both Set 1 and Set 2 and Design Fluency only in Set 2 (Figure 4 and Figure S3). Prenatal exposure to the chemical mixture in this analysis has previously been adversely associated with verbal, but not symbolic, working memory supporting the potential for the NBC exposure mixture to be more adverse for verbal than non-verbal executive function measures [61]. However, secondary analyses demonstrated an adverse association of joint exposure to the Set 1 chemical mixture with Design Fluency only among participants included in Set 2 (data not shown). These secondary findings suggest that population differences may, in part, explain differences in joint exposure associations between Set 1 and Set 2 chemical mixtures. For example, participants in Set 2 tended to have characteristics associated with more socio-economic advantage than those in Set 1.

The NBC is a prospective study with biomarkers of prenatal exposure to multiple organochlorines and metals and detailed psychometric measures of adolescent inhibition which enabled us to conduct this investigation. However, there were some limitations. First, missing data resulting from a combination of loss to follow-up and missing covariate or exposure data among those adolescents who completed psychometric testing of inhibition may result in biased estimates. We attempted to address this using IPW and results were similar to unweighted analyses suggesting that loss to follow-up bias in our findings was minimal. Furthermore, although we adjusted our analyses for maternal self-reported diet during pregnancy, this may not have been sufficient to account for the nutritional benefits of foods that are also sources of chemical exposure. The potential for residual negative confounding by diet may have resulted in underestimates of the impact of certain exposures such as PCBs and MeHg on inhibition. There are also some limitations in the use of cord blood Mn as a biomarker of Mn exposure. Mn concentrations in blood were detected by ICP-MS which detects ions based on their mass to charge ratio [65]. An isotope of Mn has a mass of 55 atomic mass units (amu) and is bordered by two isotopes of iron at 54 and 56 amu [65]. This may result in iron contributing to the Mn signal and therefore overestimating Mn concentrations among those with high iron levels [65]. However, the laboratory in which these analyses were conducted reported adequate separation of Mn and iron. Second, there is not yet consensus about which biological matrix is the most valid biomarker of Mn exposure [66,67]. However, there is evidence that cord blood Mn is a useful measure of fetal exposure and better-correlated with third-trimester dentin Mn levels than maternal biomarkers [66,68].

In conclusion, this study is among the first to estimate the association between prenatal exposures to a prevalent chemical mixture and inhibition among adolescents and provides new evidence of an adverse joint association between a chemical mixture and inhibition as measured by a verbal inhibition task. In addition, after accounting for multiple exposures, Mn appeared to be more consistently adverse than other chemicals in the mixture. Future studies assessing the impact of prenatal exposure to analogous chemical mixtures on inhibition in other populations of adolescents are needed to fully characterize the role of these prevalent exposures on critical aspects of adolescent neurodevelopment.

## Figures and Tables

**Table 1:** Characteristics of New Bedford Cohort participants who were included in the main analysis group ^1^ and those who were not included in the main analysis.

Descriptive Characteristic	NBC Participants Included in Main Analysis, *n* = 373			NBC Participants not Included in Main Analysis, *n* = 415			
**Inhibition Measures ^2^**	***n*** **(%)**	**Mean (SD)**	**Range**	***n*** **(%)**	**Mean (SD)**	**Range**	***p*** **-Value ^3^**
Design Fluency							
Total number correct scaled score	373	9.6 (2.7)	2–17	155	9.3 (3)	1–19	0.3
Total number errors raw score	373	2.2 (2.7)	0–19	155	2.6 (3.5)	0–30	0.1
Color–Word Interference scores							
Completion time scaled score	373	9.9 (2.8)	1–16	154	9.8 (2.7)	1–14	0.6
Total number errors raw score	373	2.3 (2.5)	0–19	154	2.3 (2.3)	0–12	0.7
Overall performance							
Best performance	117 (31.4)			37 (8.9)			0.1
Poor performance	256 (68.6)			117 (28.2)			
Missing	0			261 (62.9)			
**Exposure Measures ^4^**							
Cord serum DDE (ng/g)	373	0.6 (1.2)	0.02–14.9	378	0.4 (0.4)	0.0–4.2	<0.01 *
Cord serum HCB (ng/g)	373	0.03 (0.02)	0.0–0.1	378	0.03 (0.05)	0.0–0.7	0.1
Cord serum ΣPCB_4_ (ng/g)	373	0.3 (0.3)	0.01–4.4	378	0.2 (0.2)	0.01–1.9	0.05
Cord blood Pb (μg/dL)	373	1.4 (0.9)	0–9.4	375	1.7 (1.7)	0.0–17.4	<0.01 *
Cord blood Mn (µg/dL)	373	4.2 (1.6)	0.7–14.6	335	4.3 (2.0)	0.2–22.1	0.6
**Covariate Measures ^5^**							
Child Characteristics							
Race/Ethnicity							0.09
Non-Hispanic White	263 (70.5)			268 (64.6)			
Hispanic	33 (8.8)			56 (13.5)			
Other	77 (20.6)			89 (21.4)			
Missing	0			2 (0.5)			
Sex							0.05
Male	179 (48.0)			229 (55.2)			
Female	194 (52.0)			186 (44.8)			
Age at Exam	373	15.5 (0.6)	14.4–17.8	155	15.7 (0.7)	13.9–17.9	<0.01 *
Home Score	373	43.9 (6.3)	21–56	118	42.7 (6.0)	27–53	0.07
Year of birth							
1993–1994	100 (26.8)			159 (38.3)			<0.01 *
1995–1996	153 (41.0)			147 (35.4)			
1997–1998	120 (32.2)			109 (26.3)			
**Maternal Characteristics**							
Marital status at birth							<0.01 *
Not married	136 (36.5)			195 (47.0)			
Married	237 (63.5)			165 (39.8)			
Missing	0			55 (13.3)			
Maternal IQ	373	99.4 (10.4)	57–124	262	95.8 (10.2)	72–126	<0.01 *
Seafood during pregnancy (serv/day)	373	0.5 (0.6)	0–5.3	260	0.6 (0.7)	0–6	0.6
Smoking during pregnancy							0.1
No	272 (72.9)			210 (50.6)			
Yes	101 (27.1)			103 (24.8)			
Missing	0			102 (24.6)			
**Household Characteristics at Birth**							
Maternal education							<0.01 *
≤High School	190 (50.9)			231 (55.7)			
>High School	183 (49.1)			127 (30.6)			
Missing	0			57 (13.7)			
Paternal Education							<0.01 *
≤High School	246 (66.0)			266 (64.1)			
>High School	127 (34.0)			81 (19.5)			
Missing	0			68 (16.4)			
Annual Household Income							<0.01 *
<USD 20,000	115 (30.8)			150 (36.1)			
≥USD 20,000	258 (69.2)			201 (48.4)			
Missing	0			64 (15.4)			
**Examination Characteristics**							
Examiner							0.4
1	277 (74.3)			121 (29.2)			
2	96 (25.7)			34 (8.2)			
Missing	0			260 (62.7)			

^1^ Main analysis group (Set 1): complete inhibition outcome, covariate, and exposure data for, DDE, HCB, ΣPCB_4_, Pb and Mn, *n* = 373. ^2^ NBC participants with missing inhibition measures: Design Fluency total correct *n* = 260, total errors *n* = 260; Color–Word Interference completion time *n* = 261, total errors *n* = 261.^3^ P-values represent results comparing characteristics between participants included in Set 1 and those excluded from Set 1 using t-tests, Wilcoxon rank sum tests, and chi-square tests. *P*-values reflect comparisons based on non-missing data. ^4^ NBC participants with missing exposure measures: DDE *n* = 37; HCB *n* = 37; ΣPCB_4_ *n* = 37; Pb *n*= 40; Mn *n* = 80. ^5^ NBC participants with missing covariate measures: age at exam *n* = 260; HOME score *n*= 297; maternal IQ *n* = 153; seafood during pregnancy *n*= 155. * *p* < 0.05. Abbreviations: DDE: dichlorodiphenyldichloroethylene; HCB: hexachlorobenzene; ΣPCB_4_: sum of 4 PCB congeners (118, 138, 153, 180); Pb: lead; Mn: manganese.

**Table 2:** Complete-case results of multivariable linear regression analyses (difference in scaled scores associated with a twofold increase in exposure and 95% CI) ^1^ assessing the relation of prenatal exposure to a five-chemical mixture with Delis–Kaplan Executive Function System inhibition scaled scores among adolescents in the main analysis group ^2^.

Exposure	Design Fluency Total Correct Scaled Score Difference (95% CI)	Color–Word Interference Completion Time Scaled Score Difference (95% CI)
Log_2_ DDE	0.00 (−0.32, 0.33)	0.09 (−0.22, 0.40)
Log_2_ HCB	0.04 (−0.28, 0.36)	−0.08 (−0.42, 0.25)
Log_2_ ΣPCB_4_	−0.15 (−0.49, 0.18)	−0.24 (−0.59, 0.10)
Log_2_ Pb	−0.05 (−0.35, 0.26)	0.07 (−0.25, 0.39)
Log_2_ Mn	0.91 (−0.02, 1.84)	−0.74 (−1.34, −0.14) *
Log_2_ Mn^2^	−0.59 (−1.49, 0.30)	
Log_2_ DDE × Log_2_ Mn	0.53 (0.05, 1.01) *	
Log_2_ DDE × Log_2_ Mn^2^	0.03 (−0.52, 0.58)	

^1^ Exposures have been log2-transformed and models have been adjusted for all listed exposures, child race, sex, age at exam, year of birth, and HOME score; maternal marital status at child’s birth, IQ, seafood consumption during pregnancy, and smoking during pregnancy; maternal and paternal education and annual household income at child’s birth; study examiner. ^2^ Main analysis group: complete inhibition outcome, covariate and exposure data for DDE, HCB, ΣPCB_4_, Pb and Mn, *n* = 373. * *p* < 0.05. Abbreviations: DDE: dichlorodiphenyldichloroethylene; HCB: hexachlorobenzene; ΣPCB_4_: sum of 4 PCB congeners (118, 138, 153, 180); Pb: lead; Mn: manganese.

**Table 3:** Sex-stratified results of multivariable linear regression analyses (difference in scaled scores associated with a twofold increase in exposure and 95% CI) ^1^ assessing the relation of prenatal exposure to a five-chemical mixture with Delis–Kaplan Executive Function System inhibition scaled scores among adolescents in the main analysis group ^2^.

	Design Fluency Total Correct Scaled Score			Color–Word Interference Completion Time Scaled Score		
Exposure	Males Difference (95% CI)	Females Difference (95% CI)	*p* ^3^	Males Difference (95% CI)	Females Difference (95% CI)	*p* ^3^
Log_2_ DDE	−0.18 (−0.70, 0.34)	−0.17 (−0.66, 0.32)	0.7	0.00 (−0.47, 0.47)	0.25 (−0.23, 0.72)	0.4
Log_2_ HCB	−0.19 (−0.64, 0.27)	0.34 (−0.12, 0.81)	0.1	−0.03 (−0.54, 0.48)	−0.11 (−0.59, 0.38)	0.8
Log_2_ ΣPCB_4_	0.04 (−0.45, 0.53)	−0.25 (−0.78, 0.28)	1.0	−0.09 (−0.60, 0.43)	−0.47 (−0.99, 0.04)	0.5
Log_2_ Pb	0.28 (−0.25, 0.81)	−0.25 (−0.63, 0.13)	0.4	0.20 (−0.39, 0.79)	−0.05 (−0.44, 0.33)	0.5
Log_2_ Mn	1.90 (0.47, 3.34) *	−0.06 (−1.47, 1.36)	0.1	−0.80 (−1.69, 0.09)	−0.48 (−1.34, 0.37)	0.5
Log_2_ Mn^2^	0.07 (−1.22, 1.36)	−0.27 (−1.67, 1.12)	0.3			
Log_2_ DDE × Log_2_ Mn	0.83 (0.06, 1.60) *	0.10 (−0.64, 0.84)	0.2			
Log_2_ DDE × Log_2_ Mn^2^	0.49 (−0.37, 1.35)	0.03 (−0.74, 0.80)	0.3			

^1^ Exposures have been log2-transformed and models have been adjusted for all listed exposures, child race, sex, age at exam, year of birth, and HOME score; maternal marital status at child’s birth, IQ, seafood consumption during pregnancy, and smoking during pregnancy; maternal and paternal education and annual household income at child’s birth; study examiner. ^2^ Main analysis group: complete inhibition outcome, covariate and exposure data for DDE, HCB, ΣPCB_4,_ Pb and Mn, total *n* = 373; males *n*= 179; females *n* = 194. ^3^ *p*-value for chemical-sex interaction term included in multivariable linear regression model. * *p* < 0.05. Abbreviations: DDE: dichlorodiphenyldichloroethylene; HCB: hexachlorobenzene; ΣPCB_4_: Sum of 4 PCB congeners (118, 138, 153, 180); Pb: lead; Mn: manganese.

**Table 4:** Prenatal social disadvantage index (PNSDI) ^1^-stratified results of multivariable linear regression analyses (difference in scaled scores associated with a twofold increase in exposure and 95% CI) ^2^ assessing the relation of prenatal exposure to a five-chemical mixture with Delis–Kaplan Executive Function System inhibition scaled scores among adolescents in the main analysis group ^3^.

	Design Fluency Total Correct Scaled Score			Color–Word Interference Completion Time Scaled Score		
Exposure	PNSDI < 3 Difference (95% CI)	PNSDI ≥ 3 Difference (95% CI)	*p* ^4^	PNSDI < 3 Difference (95% CI)	PNSDI ≥ 3 Difference (95% CI)	*p* ^4^
Log_2_ DDE	−0.07 (−0.48, 0.35)	−0.03 (−0.60, 0.53)	0.5	0.26 (−0.12, 0.64)	−0.18 (−0.79, 0.43)	0.3
Log_2_ HCB	0.31 (−0.09, 0.72)	−0.57 (−1.13, 0.00)	0.01 *	−0.01 (−0.43, 0.42)	−0.26 (−0.86, 0.34)	0.2
Log_2_ ΣPCB_4_	−0.23 (−0.63, 0.17)	0.21 (−0.47, 0.90)	0.2	−0.40 (−0.82, 0.01)	0.15 (−0.52, 0.83)	0.2
Log_2_ Pb	−0.32 (−0.73, 0.08)	0.27 (−0.26, 0.80)	0.1	0.01 (−0.40, 0.43)	0.07 (−0.51, 0.65)	1.0
Log_2_ Mn	1.28 (0.12, 2.45) *	1.26 (−1.34, 3.87)	1.0	−0.76 (−1.52, 0.00)	−0.69 (−1.76, 0.39)	0.9
Log_2_ Mn^2^	0.44 (−0.77, 1.65)	−2.00 (−4.29, 0.30)	0.1			
Log_2_ DDE × Log_2_ Mn	0.47 (−0.14, 1.08)	0.81 (−0.38, 1.99)	0.8			
Log_2_ DDE × Log_2_ Mn^2^	0.39 (−0.36, 1.15)	−0.24 (−1.30, 0.82)	0.4			

^1^ Prenatal social disadvantage index (PNSDI) was constructed as the sum of five adverse social or economic exposures at the time of the child’s birth where presence of each risk factor was assigned a value of 1, absence a value of 0: mother unmarried, mother’s education as high school graduate or less, father’s education as high school graduate or less, annual household income less than USD 20,000, and mother’s age at birth less than 20 years. ^2^ Exposures have been log2-transformed and models have been adjusted for all listed exposures, child race, sex, age at exam, year of birth, and HOME score; maternal marital status at child’s birth, IQ, seafood consumption during pregnancy, and smoking during pregnancy; maternal and paternal education and annual household income at child’s birth; study examiner. ^3^ Main analysis group: complete inhibition outcome, covariate and exposure data for DDE, HCB, ΣPCB_4_, Pb, and Mn, *n* = 373. Total *n* = 373; PNSDI < 3 *n*= 241; PNSDI ≥ 3 *n* = 132. ^4^ *p*-value for chemical-PNSDI interaction term included in multivariable linear regression model. * *p* < 0.05. Abbreviations: DDE: dichlorodiphenyldichloroethylene; HCB: hexachlorobenzene; ΣPCB_4_: sum of 4 PCB congeners (118, 138, 153, 180); Pb: lead; Mn: manganese.

**Table 5:** Complete-case results of negative binomial regression analyses (rate ratio and 95% CI) ^1^ assessing the relation of prenatal exposure to a five-chemical mixture with Delis–Kaplan Executive Function System (D-KEFS) inhibition error raw scores among adolescents in the main analysis group ^2^.

Exposure	Design Fluency Total Errors Rate Ratio (95% CI)	Color–Word Interference Total Errors Rate Ratio (95% CI)
Log_2_ DDE	1.04 (0.92, 1.17)	0.91 (0.82, 1.02)
Log_2_ HCB	1.08 (0.95, 1.24)	1.07 (0.95, 1.21)
Log_2_ ΣPCB_4_	0.94 (0.82, 1.08)	1.13 (1.00, 1.28)
Log_2_ Pb	1.00 (0.88, 1.13)	0.91 (0.82, 1.02)
Log_2_ Mn	0.78 (0.61, 0.99) *	1.10 (0.88, 1.36)

^1^ Exposures have been log2-transformed and models have been adjusted for child race, sex, age at exam, year of birth, and HOME score; maternal marital status at child’s birth, IQ, seafood consumption during pregnancy, and smoking during pregnancy; maternal and paternal education and annual household income at child’s birth; and study examiner. ^2^ Main analysis group: complete inhibition outcome, covariate and prenatal exposure biomarker data for DDE, HCB, ΣPCB_4_, Pb and Mn, *n* = 373. * *p* < 0.05. Abbreviations: DDE: dichlorodiphenyldichloroethylene; HCB: hexachlorobenzene; ΣPCB_4_: sum of 4 PCB congeners (118, 138, 153, 180); Pb: lead; Mn: manganese.

**Table 6:** Complete-case results of logistic regression analyses (odds ratio and 95% CI) ^1^ assessing the relation of prenatal exposure to a five-chemical mixture with odds of poor Delis–Kaplan Executive Function System (D-KEFS) Color–Word Interference: Inhibition overall performance among adolescents in the main analysis group ^2^.

Exposure	Color–Word Interference Overall Performance Odds Ratio (95% CI)
	Best performance: *n* = 117
	Poor performance: *n* = 256
Log_2_ DDE	0.93 (0.70, 1.22)
Log_2_ HCB	1.22 (0.93, 1.60)
Log_2_ ΣPCB_4_	1.16 (0.87, 1.55)
Log_2_ Pb	0.79 (0.59, 1.05)
Log_2_ Mn	1.61 (0.98, 2.64)

^1^ Exposures have been log2-transformed and models have been adjusted for child race, sex, age at exam, year of birth, and HOME score; maternal marital status at child’s birth, IQ, seafood consumption during pregnancy, and smoking during pregnancy; maternal and paternal education and annual household income at child’s birth; and study examiner. ^2^ Main analysis group: complete outcome, covariate and prenatal exposure biomarker data for DDE, HCB, ΣPCB_4,_ Pb and Mn, *n* = 373. Abbreviations: DDE: dichlorodiphenyldichloroethylene; HCB: hexachlorobenzene; ΣPCB_4_: sum of 4 PCB congeners (118, 138, 153, 180); Pb: lead; Mn: manganese.

## Data Availability

The data are not publicly available due to privacy and confidentiality reasons.

## Supplementary Materials

The following are available online at https://www.mdpi.com/article/10.3390/toxics9110311/s1, Figure S1: Estimated exposure–response functions and 95% credible intervals of each of the seven exposures in Set 2 with the Delis–Kaplan Executive Function System inhibition scale scores, where all remaining exposures are assigned to their median value among adolescents in the secondary analysis group, Figure S2: Exposure–response functions associating each of the seven exposures (Set 2) and a second exposure fixed at various quantiles with the Delis–Kaplan Executive Function System inhibition scaled scores, while the remaining exposures are assigned to their median value among adolescents in the secondary analysis group, Figure S3: Joint association (estimates and 95% credible intervals) of the seven-chemical mixture (DDE, HCB, ΣPCB_4_, Pb, Mn, MeHg, As) with the Delis–Kaplan Executive Function System inhibition scaled score among adolescents in the secondary analysis group (Set 2). Chemical mixture levels at each percentile are compared to each component at its median level, Table S1: Characteristics of New Bedford Cohort participants who were included in the secondary analysis group (with a seven-chemical exposure mixture) and those who were excluded from the secondary analysis group. Table S2: Inverse probability weighted results of multivariable linear regression analyses (difference in scaled scores associated with a twofold increase in exposure and 95% CI) assessing the relation of prenatal exposure to a five-chemical mixture with Delis–Kaplan Executive Function System inhibition scaled scores among adolescents in the main analysis group, Table S3: Inverse probability weighted sex-stratified results of multivariable linear regression analyses (difference in scaled scores associated with a twofold increase in exposure and 95% CI) assessing the relation of prenatal exposure to a five-chemical mixture with Delis–Kaplan Executive Function System inhibition scaled scores among adolescents in the main analysis group, Table S4: Inverse probability weighted prenatal social disadvantage index (PNSDI)-stratified results of multivariable linear regression analyses (difference in scaled scores associated with a twofold increase in exposure and 95% CI) assessing the relation of prenatal exposure to a five-chemical mixture with Delis–Kaplan Executive Function System inhibition scaled scores among adolescents in the main analysis group, Table S5: Inverse probability weighted results of negative binomial regression analyses (rate ratio and 95% CI) assessing the relation of prenatal exposure to a five-chemical mixture with Delis–Kaplan Executive Function System (D-KEFS) inhibition error raw scores among adolescents in the main analysis group, Table S6: Inverse probability weighted results of logistic regression analyses (odds ratio and 95% CI) assessing the relation of prenatal exposure to a five-chemical mixture with Delis–Kaplan Executive Function System (D-KEFS) Color–Word Interference: Inhibition overall performance among adolescents in the main analysis group, Table S7: Complete-case results of multivariable linear regression analyses (difference in scaled scores associated with a twofold increase in exposure and 95% CI) assessing the relation of prenatal exposure to a seven-chemical mixture with Delis–Kaplan Executive Function System inhibition scaled scores among adolescents in the secondary analysis group, Table S8: Inverse probability weighted results of multivariable linear regression analyses (difference in scaled scores associated with a twofold increase in exposure and 95% CI) assessing the relation of prenatal exposure to a seven-chemical mixture with Delis–Kaplan Executive Function System inhibition scaled scores among adolescents in the secondary analysis group.
