# Supplementary material for: Prenatal Exposure to Chemical Mixtures and Inhibition among Adolescents
Source: Toxics. 2021 Nov 16;9(11):311. doi: 10.3390/toxics9110311 (PMC8619850; doi:10.3390/toxics9110311)
Supplement: Supplementary file 1 [file toxics-09-00311-s001.zip › toxics-1437910-supplementary.pdf]

toxics

MDPI

Supplementary Materials:

Prenatal Exposure to Chemical Mixtures and Inhibition among Adolescents

Anna V. Oppenheimer, David C. Bellinger, Brent A. Coull, Marc G. Weisskopf, Michele Zemplenyi and Susan A. Korrick

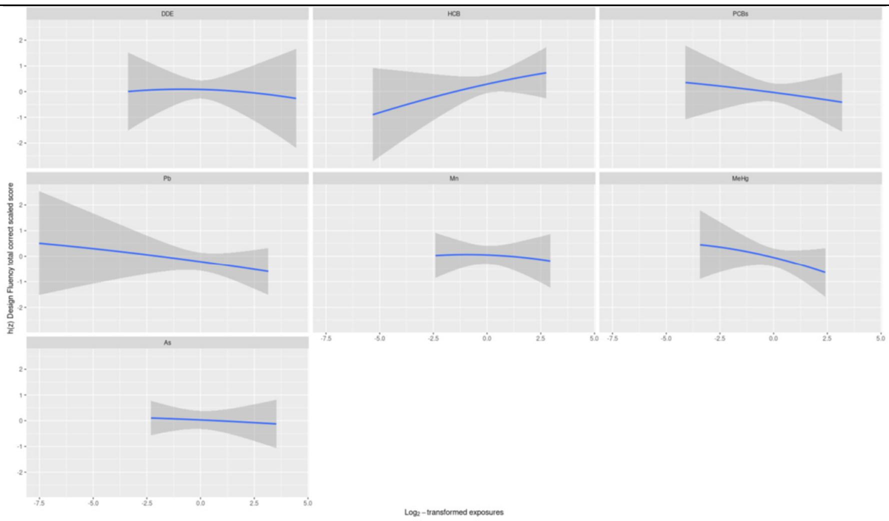

(**a**) Design Fluency total correct scaled score

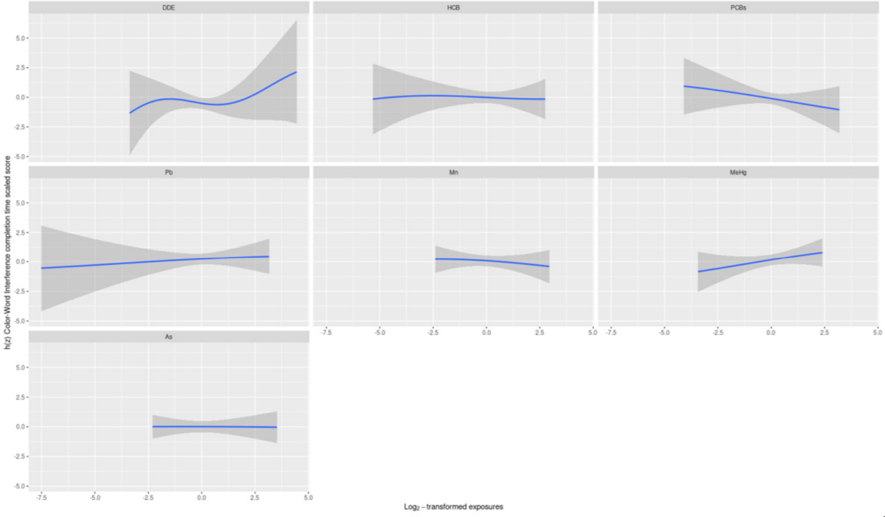

(**b**) Color-Word Interference completion time scaled score

Figure S1. Estimated exposure–response functions and 95% credible intervals[1] of each of the seven exposures in Set 2[2] with the Delis–Kaplan Executive Function System inhibition scale scores, where all remaining exposures are assigned to their median value among adolescents in the secondary analysis group.

[1]Exposures have been log2–transformed and models have been adjusted for child race, sex, age at exam, year of birth and HOME score; maternal marital status at child's birth, IQ, seafood consumption during pregnancy, and smoking during pregnancy; maternal and paternal education and annual household income at child's birth; and study examiner. [2]Set 2: complete outcome, covariate and exposure data for PCBs, DDE, HCB, Pb, Mn, MeHg, and As, n = 235.Abbreviations: DDE: dichlorodiphenyldichloroethylene; HCB: hexachlorobenzene; PCBs: Sum of 4 PCB congeners (118, 138, 153, 180); Pb: lead; Mn: manganese, MeHg: methylmercury; As: arsenic.

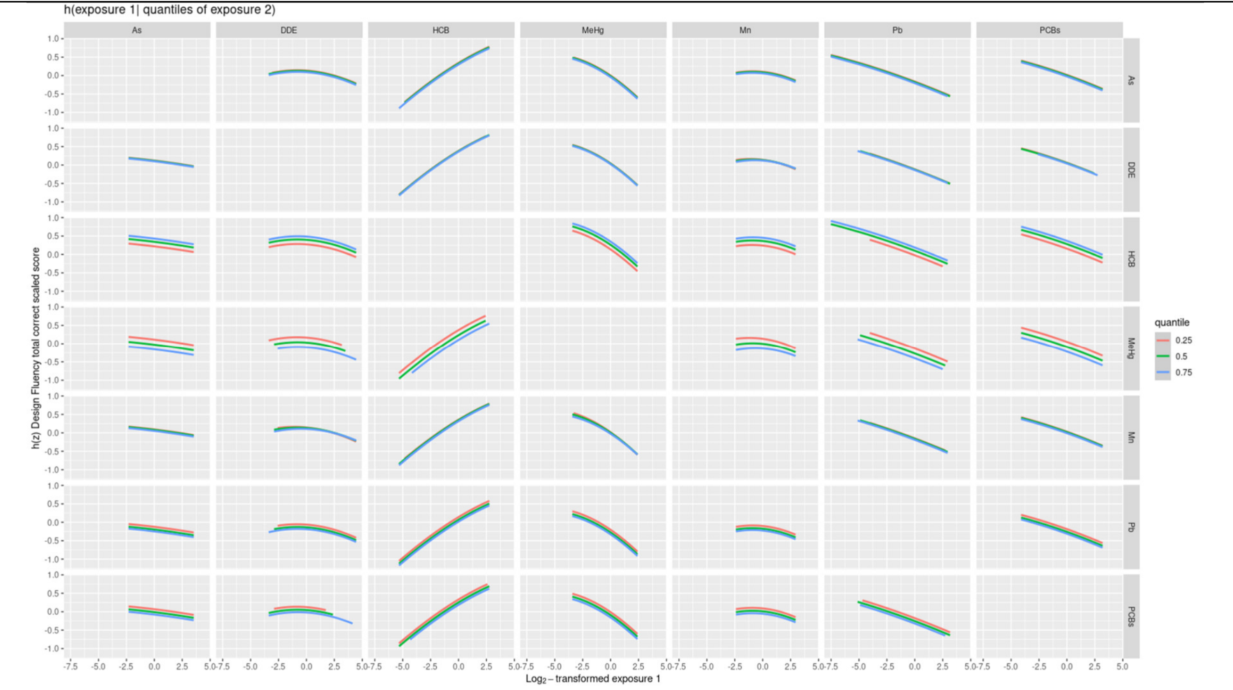

(**a**) Design Fluency total correct scaled score

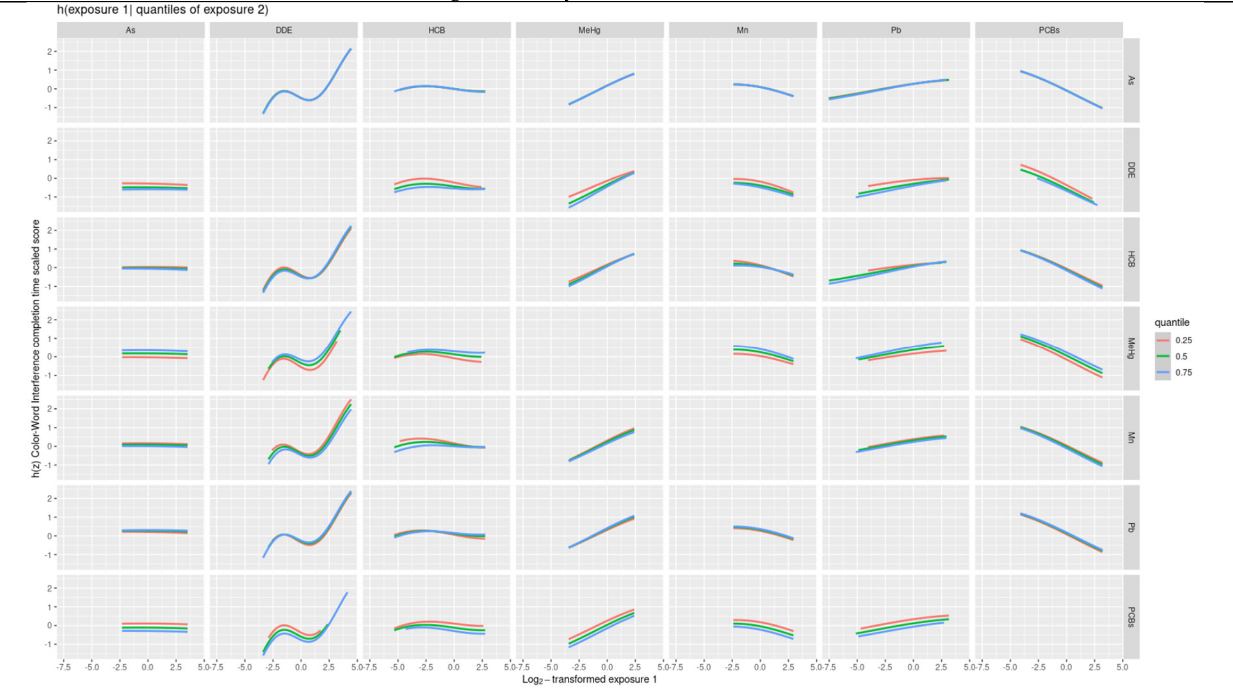

(**b**) Color-Word Interference completion time scaled score

Figure S2. Exposure–response functions[1] associating each of the seven exposures (Set 2[2]) and a second exposure fixed at various quantiles with the Delis Kaplan Executive Function System inhibition scaled scores, while the remaining exposures are assigned to their median value among adolescents in the secondary analysis group.

[1]Exposures have been log2–transformed and models have been adjusted for child race, sex, age at exam, year of birth, and HOME score; maternal marital status at child's birth, IQ, seafood consumption during pregnancy, and smoking during pregnancy; maternal and paternal education and annual household income at child's birth; and study examiner. [2]Set 2: complete inhibition outcome, covariate and exposure data for PCBs, DDE, HCB, Pb, Mn, MeHg, and As, *n* = 235. Abbreviations: DDE: dichlorodiphenyldichloroethylene; HCB: hexachlorobenzene; ΣPCB_4: Sum of 4 PCB congeners (118, 138, 153, 180); Pb: lead; Mn: manganese; MeHg: methylmercury; As: arsenic.

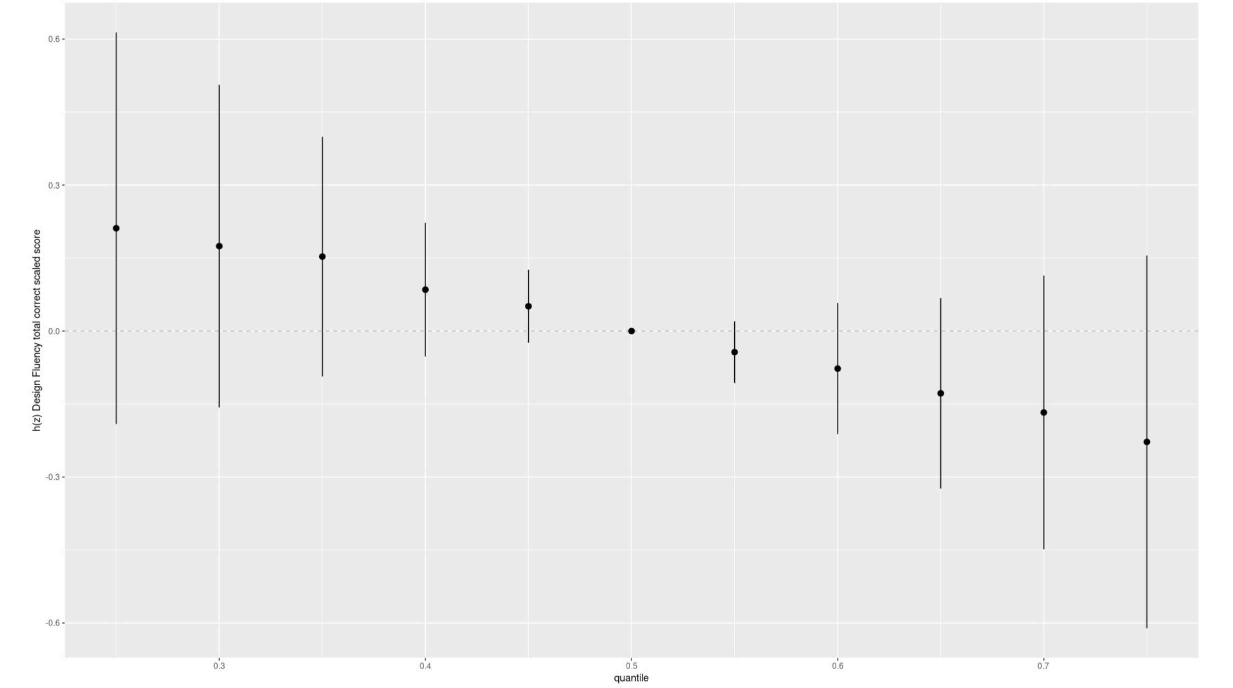

(**a**) Design Fluency total correct scaled score

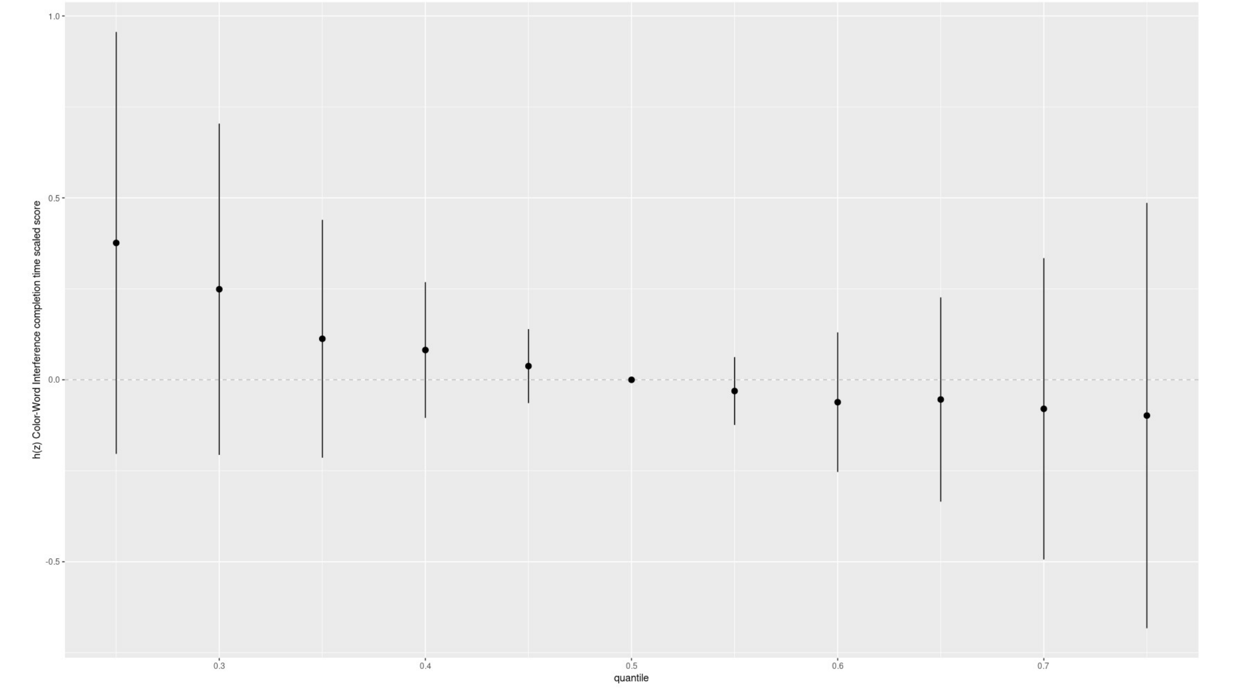

(**b**) Color-Word Interference completion time scaled score

Figure S3. Joint association (estimates and 95% credible intervals)[1] of the seven–chemical mixture (DDE, HCB, ΣPCB$_4$, Pb, Mn, MeHg, As) with the Delis–Kaplan Executive Function System inhibition scaled score among adolescents in the secondary analysis group (Set 2)[2]. Chemical mixture levels at each percentile are compared to a mixture with each component at its median level.

[1]Exposures have been log2–transformed and models have been adjusted for child race, sex, age at exam, year of birth, and HOME score; maternal marital status at child's birth, IQ, seafood consumption during pregnancy, and smoking during pregnancy; maternal and paternal education and annual household income at child's birth; and study examiner. [2]Set 2: complete inhibition outcome, covariate and exposure data for PCBs, DDE, HCB, Pb, Mn, MeHg, and As, *n* = 235. Abbreviations: DDE: dichlorodiphenyldichloroethylene; HCB: hexachlorobenzene; ΣPCB$_4$: Sum of 4 PCB congeners (118, 138, 153, 180); Pb: lead; Mn: manganese: MeHg: methylmercury; As: arsenic.

Table S1. Characteristics of New Bedford Cohort participants included in the secondary analysis group (with a seven chemical exposure mixture)[1] and those who were excluded from the secondary analysis group.

Descriptive Characteristic	Main analysis group, *n* = 235			Excluded group, *n* = 553			
**Inhibition Measures**[2]	***n*** **(%)**	**Mean (SD)**	**Range**	***n*** **(%)**	**Mean (SD)**	**Range**	***p*–value**[3]
Design Fluency							
Total number correct scaled score	235	9.8 (2.6)	2–17	293	9.2 (2.9)	1–19	0.01*
Total number errors raw score	235	2.2 (2.6)	0–18	293	2.4 (3.2)	0–30	0.4
Color–Word Interference scores							
Completion time scaled score	235	10.2 (2.8)	1–16	292	9.7 (2.8)	1–14	0.03*
Total number errors raw score	235	2.1 (2.2)	0–11	292	2.5 (2.5)	0–19	0.02*
Overall performance							
Best performance	88 (37.4)			66 (11.9)			< 0.01*
Poor performance	147 (62.6)			226 (40.9)			
Missing	0			261 (47.2)			
**Exposure Measures**[4]							
Cord serum DDE (ng/g)	235	0.6 (1.4)	0.02–14.9	516	0.4 (0.7)	0–10.2	0.04*
Cord serum HCB (ng/g)	235	0.03 (0.02)	0–0.1	516	0.03 (0.04)	0–0.7	0.2
Cord serum ΣPCB_4 (ng/g)	235	0.3 (0.3)	0.01–2.3	516	0.2 (0.3)	0.01–4.4	0.2
Cord blood Pb (μg/dL)	235	1.4 (0.9)	0–9.4	513	1.6 (1.5)	0.01–17.4	0.01*
Cord blood Mn (μg/dL)	235	4.3 (1.6)	1.7–11.2	473	4.2 (1.9)	0.2–22.1	0.8
Maternal hair total Hg (μg/g)	235	0.6 (0.6)	0.03–3.1	276	0.6 (0.7)	0.03–9.2	0.3
Maternal toenail As (μg/g)	235	0.1 (0.1)	0.02–0.8	181	0.1 (0.1)	0.02–1.0	0.5
**Covariate Measures**[5]							
Child Characteristics							
Race/Ethnicity							< 0.01*
Non–Hispanic White	186 (79.1)			345 (62.4)			
Hispanic	16 (6.8)			73 (13.2)			
Other	33 (14.0)			133 (24.1)			
Missing	0			2 (0.4)			
Sex							0.3
Male	114 (48.5)			294 (53.2)			
Female	121 (51.5)			259 (46.8)			
Age at Exam	235	15.5 (0.6)	14.4–17.7	293	15.6 (0.6)	13.9–17.9	0.5
Home Score	235	44.4 (6.0)	27–56	256	42.8 (6.5)	21–56	< 0.01*
Year of birth							0.03*
1993–1994	76 (32.3)			183 (33.1)			
1995–1996	104 (44.3)			196 (35.4)			
1997–1998	55 (23.4)			174 (31.5)			
Maternal Characteristics							
Marital status at birth							< 0.01*
Not married	74 (31.5)			257 (46.5)			
Married	161 (68.5)			241 (43.6)			
Missing	0			55 (9.9)			
Maternal IQ	235	100.6 (9.7)	67–124	400	96.3 (10.5)	57–126	< 0.01*
Seafood during pregnancy (serv/day)	235	0.5 (0.6)	0–5.3	398	0.6 (0.7)	0–6	0.5
Smoking during pregnancy							0.3
No	171 (72.8)			311 (56.2)			
Yes	64 (27.2)			140 (25.3)			
Missing	0			102 (18.4)			
Household Characteristics at Birth							
Maternal education							< 0.01*
≤ High School	108 (46.0)			313 (56.6)			
> High School	127 (54.0)			183 (33.1)			
Missing	0			57 (10.3)			
Paternal Education							0.01*

≤ High School	152 (64.7)	360 (65.1)	
> High School	83 (35.3)	125 (22.6)	
Missing	0	68 (12.3)	
Annual Household Income			< 0.01*
< $20,000	62 (26.4)	203 (36.7)	
≥ $20,000	173 (73.6)	286 (51.7)	
Missing	0	64 (11.6)	
Examination Characteristics			
Examiner			0.3
1	171 (72.8)	227 (41.0)	
2	64 (27.2)	66 (11.9)	
Missing	0	260 (47.0)	

[1]Secondary analysis group (Set 2): complete inhibition outcome, covariate, and exposure data for, DDE, HCB, ΣPCB_4, Pb, Mn, MeHg, and As, $n = 235$. [2]NBC participants with missing inhibition measures: Design Fluency total correct n=260, total errors $n = 260$; Color–Word Interference completion time n=261, total errors n=261. [3]p–values represent results comparing characteristics between participants included in Set 2 and those excluded from Set 2 using t–tests, Wilcoxon rank sum tests, and chi–square tests. p–values reflect comparisons based on non–missing data. [4]NBC participants with missing exposure measures: DDE $n = 37$; HCB $n = 37$; ΣPCB_4 $n = 37$; Pb $n = 40$; Mn $n = 80$; MeHg $n = 277$; As $n = 372$. [5]NBC participants with missing covariate measures: age at exam $n = 260$; HOME score $n = 297$; maternal IQ $n = 153$; seafood during pregnancy $n = 155$. *$p < 0.05$. Abbreviations: DDE: dichlorodiphenyldichloroethylene; HCB: hexachlorobenzene; ΣPCB_4: Sum of 4 PCB congeners (118, 138, 153, 180); Pb: lead; Mn: manganese; Hg: mercury; As: arsenic.

Table S2. Inverse probability weighted results of multivariable linear regression analyses (difference in scaled scores associated with a twofold increase in exposure and 95% CI)[1] assessing the relation of prenatal exposure to a five–chemical mixture with Delis Kaplan Executive Function System inhibition scaled scores among adolescents in the main analysis group[2].

Exposure	Design Fluency total correct scaled score Difference (95% CI)	Color–Word Interference completion time scaled score Difference (95% CI)
Log_2 DDE	0.01 (–0.31, 0.33)	0.07 (–0.24, 0.38)
Log_2 HCB	–0.01 (–0.33, 0.31)	–0.08 (–0.42, 0.26)
Log_2 ΣPCB_4	–0.15 (–0.48, 0.18)	–0.22 (–0.56, 0.12)
Log_2 Pb	0.02 (–0.29, 0.33)	0.11 (–0.21, 0.44)
Log_2 Mn	0.87 (–0.05, 1.79)	–0.84 (–1.44, –0.23) *
Log_2 Mn^2	–0.67 (–1.53, 0.19)	
Log_2 DDE × Log_2 Mn	0.54 (0.06, 1.01) *	
Log_2 DDE × Log_2 Mn^2	0.05 (–0.47, 0.57)	

[1]Exposures have been log2–transformed and models have been adjusted for all listed exposures, child race, sex, age at exam, year of birth, and HOME score; maternal marital status at child's birth, IQ, seafood consumption during pregnancy, and smoking during pregnancy; maternal and paternal education and annual household income at child's birth; study examiner. [2]Main analysis group: complete inhibition outcome, covariate and exposure data for PCBs, DDE, HCB, Pb and Mn. [2]Total $n = 373$. *$p < 0.05$. Abbreviations: DDE: dichlorodiphenyldichloroethylene; HCB: hexachlorobenzene; ΣPCB_4: Sum of 4 PCB congeners (118, 138, 153, 180); Pb: lead; Mn: manganese.

Table S3. Inverse probability weighted sex–stratified results of multivariable linear regression analyses (difference in scaled scores associated with a twofold increase in exposure and 95% CI)[1] assessing the relation of prenatal exposure to a five–chemical mixture with Delis Kaplan Executive Function System inhibition scaled scores among adolescents in the main analysis group[2].

	Design Fluency total correct scaled score			Color–Word Interference completion time scaled score		
**Exposure**	**Males Difference (95% CI)**	**Females Difference (95% CI)**	p^3	**Males Difference (95% CI)**	**Females Difference (95% CI)**	p^3
Log_2 DDE	–0.23 (–0.75, 0.29)	–0.15 (–0.63, 0.34)	0.5	–0.02 (–0.49, 0.45)	0.24 (–0.23, 0.72)	0.4
Log_2 HCB	–0.28 (–0.73, 0.17)	0.37 (–0.10, 0.84)	0.1	–0.02 (–0.53, 0.49)	–0.12 (–0.60, 0.37)	0.8
Log_2 ΣPCB_4	0.03 (–0.45, 0.52)	–0.24 (–0.77, 0.28)	1.0	–0.10 (–0.62, 0.42)	–0.46 (–0.97, 0.04)	0.5
Log_2 Pb	0.47 (–0.06, 1.00)	–0.25 (–0.63, 0.13)	0.2	0.25 (–0.35, 0.84)	–0.03 (–0.41, 0.36)	0.6
Log_2 Mn	1.81 (0.39, 3.23) *	–0.10 (–1.48, 1.28)	0.1	–0.86 (–1.76, 0.04)	–0.58 (–1.43, 0.27)	0.5
Log_2 Mn^2	0.09 (–1.20, 1.39)	–0.19 (–1.46, 1.08)	0.5			
Log_2 DDE × Log_2 Mn	0.84 (0.10, 1.59) *	0.08 (–0.64, 0.80)	0.2			
Log_2 DDE × Log_2 Mn^2	0.54 (–0.28, 1.37)	0.06 (–0.68, 0.79)	0.2			

[1]Exposures have been log2–transformed and models have been adjusted for all listed exposures, child race, sex, age at exam, year of birth, and HOME score; maternal marital status at child's birth, IQ, seafood consumption during pregnancy, and smoking during pregnancy; maternal and paternal education and annual household income at child's birth; study examiner. [2]Main analysis group: complete inhibition outcome, covariate and exposure data for PCBs, DDE, HCB, Pb and Mn. Total $n = 373$; Males $n = 179$; Females $n = 194$. [3]p–value for chemical–sex interaction term included in multivariable linear regression model. *$p < 0.05$. Abbreviations: DDE: dichlorodiphenyldichloroethylene; HCB: hexachlorobenzene; ΣPCB_4: Sum of 4 PCB congeners (118, 138, 153, 180); Pb: lead; Mn: manganese.

Table S4. Inverse probability weighted prenatal social disadvantage index (PNSDI)[1]–stratified results of multivariable linear regression analyses (difference in scaled scores associated with a twofold increase in exposure and 95% CI)[2] assessing the relation of prenatal exposure to a five–chemical mixture with Delis–Kaplan Executive Function System inhibition scaled scores among adolescents in the main analysis group[3].

	**Design Fluency total correct scaled score**			**Color–Word Interference completion time scaled score**		
**Exposure**	**PNSDI < 3 Difference (95% CI)**	**PNSDI ≥ 3 Difference (95% CI)**	p^4	**PNSDI < 3 Difference (95% CI)**	**PNSDI ≥ 3 Difference (95% CI)**	p^4
Log_2 DDE	–0.07 (–0.48, 0.34)	–0.06 (–0.62, 0.50)	0.5	0.28 (–0.09, 0.64)	–0.27 (–0.88, 0.34)	0.3
Log_2 HCB	0.30 (–0.10, 0.70)	–0.62 (–1.17, –0.08)*	0.01*	0.02 (–0.40, 0.45)	–0.20 (–0.78, 0.39)	0.3
Log_2 ΣPCB_4	–0.21 (–0.60, 0.19)	0.17 (–0.51, 0.85)	0.3	–0.40 (–0.81, 0.00)	0.15 (–0.51, 0.81)	0.2
Log_2 Pb	–0.34 (–0.74, 0.06)	0.39 (–0.15, 0.92)	0.1	0.01 (–0.40, 0.43)	0.15 (–0.43, 0.74)	0.9
Log_2 Mn	1.31 (0.20, 2.42)*	1.54 (–1.02, 4.09)	1.0	–0.98 (–1.73, –0.22)*	–0.73 (–1.79, 0.33)	1.0
Log_2 Mn^2	0.52 (–0.64, 1.68)	–2.25 (–4.43, –0.06) *	0.1			
Log_2 DDE × Log_2 Mn	0.46 (–0.13, 1.04)	0.93 (–0.23, 2.09)	0.7			
Log_2 DDE × Log_2 Mn^2	0.45 (–0.26, 1.17)	–0.29 (–1.30, 0.71)	0.4			

[1]Prenatal social disadvantage index (PNSDI) was constructed as the sum of five adverse social or economic exposures at the time of the child's birth where presence of each risk factor was assigned a value of 1, absence a value of 0: mother unmarried, mother's education as high school graduate or less, father's education as high school graduate or less, annual household income less than USD 20,000, and mother's age at birth less than 20 years. [2]Exposures have been log2–transformed and models have been adjusted for all listed exposures, child race, sex, age at exam, year of birth, and HOME score; maternal marital status at child's birth, IQ, seafood consumption during pregnancy, and smoking during pregnancy; maternal and paternal education and annual household income at child's birth; study examiner. [3]Main analysis group: complete inhibition outcome, covariate and exposure data for PCBs, DDE, HCB, Pb and Mn. Total $n = 373$; PNSDI < 3 $n = 241$; PNSDI ≥ 3 $n = 132$. [4]*P*–value for chemical–PNSDI interaction term included in multivariable linear regression model. *$p < 0.05$. Abbreviations: DDE: dichlorodiphenyldichloroethylene; HCB: hexachlorobenzene; ΣPCB_4: Sum of 4 PCB congeners (118, 138, 153, 180); Pb: lead; Mn: manganese.

Table S5. Inverse probability weighted results of negative binomial regression analyses (rate ratio and 95% CI)[1] assessing the relation of prenatal exposure to a five– chemical mixture with Delis–Kaplan Executive Function System (D–KEFS) inhibition error raw scores among adolescents in the main analysis group[2].

Exposure	Design Fluency total errors<br>Rate ratio (95% CI)	Color–Word Interference total errors<br>Rate ratio (95% CI)
Log_2 DDE	1.04 (0.93, 1.17)	0.92 (0.83, 1.03)
Log_2 HCB	1.09 (0.96, 1.25)	1.07 (0.95, 1.21)
Log_2 ΣPCB_4	0.94 (0.82, 1.07)	1.11 (0.98, 1.25)
Log_2 Pb	1.01 (0.89, 1.14)	0.92 (0.83, 1.03)
Log_2 Mn	0.73 (0.58, 0.92) *	1.09 (0.88, 1.34)

[1]Exposures have been log2–transformed and models have been adjusted for child race, sex, age at exam, year of birth, and HOME score; maternal marital status at child's birth, IQ, seafood consumption during pregnancy, and smoking during pregnancy; maternal and paternal education and annual household income at child's birth; and study examiner.[2]Main analysis group: complete inhibition outcome, covariate and prenatal exposure biomarker data for DDE, HCB, ΣPCB_4, Pb and Mn, $n = 373$. * $p < 0.05$. Abbreviations: DDE: dichlorodiphenyldichloroethylene; HCB: hexachlorobenzene; ΣPCB_4: Sum of 4 PCB congeners (118, 138, 153, 180); Pb: lead; Mn: manganese.

Table S6. Inverse probability weighted results of logistic regression analyses (odds ratio and 95% CI)[1] assessing the relation of prenatal exposure to a five– chemical mixture with Delis–Kaplan Executive Function System (D–KEFS) Color–Word Interference: Inhibition overall performance[2] among adolescents in the main analysis group[3].

Exposure	Color–Word Interference overall performance<br>Odds ratio (95% CI)
	Best performance: n = 117<br>*Poor performance: n = 256*
Log_2 DDE	0.92 (0.70, 1.21)
Log_2 HCB	1.24 (0.94, 1.64)
Log_2 ΣPCB_4	1.15 (0.86, 1.53)
Log_2 Pb	0.77 (0.58, 1.04)
Log_2 Mn	1.69 (1.02, 2.81) *

[1]Exposures have been log2–transformed and models have been adjusted for child race, sex, age at exam, year of birth, and HOME score; maternal marital status at child's birth, IQ, seafood consumption during pregnancy, and smoking during pregnancy; maternal and paternal education and annual household income at child's birth; and study examiner. [2]Reference is best performance group (total completion time raw score < median and total errors raw score < median) compared to remaining participants (poor performance group). . [3]Main analysis group: complete inhibition outcome, covariate and prenatal exposure biomarker data for DDE, HCB, ΣPCB_4, Pb and Mn, *n* = 373. *$p < 0.05$. Abbreviations: DDE: dichlorodiphenyldichloroethylene; HCB: hexachlorobenzene; ΣPCB_4: Sum of 4 PCB congeners (118, 138, 153, 180); Pb: lead; Mn: manganese.

Table S7. Complete-case results of multivariable linear regression analyses (difference in scaled scores associated with a twofold increase in exposure and 95% CI)[1] assessing the relation of prenatal exposure to a seven–chemical mixture with Delis Kaplan Executive Function System inhibition scaled scores among adolescents in the secondary analysis group[2].

Exposure	Design Fluency total correct scaled score Difference (95% CI)	Color–Word Interference completion time scaled score Difference (95% CI)
Log_2 DDE	0.02 (–0.40, 0.43)	0.32 (–0.11, 0.75)
Log_2 HCB	0.29 (–0.11, 0.69)	0.03 (–0.38, 0.44)
Log_2 ΣPCB_4	–0.23 (–0.70, 0.24)	–0.54 (–1.02, –0.06) *
Log_2 Pb	–0.27 (–0.67, 0.12)	0.07 (–0.34, 0.47)
Log_2 Mn	–0.12 (–0.84, 0.61)	–0.64 (–1.38, 0.11)
Log_2 MeHg	–0.20 (–0.56, 0.17)	0.36 (–0.02, 0.73)
Log_2 As	–0.06 (–0.41, 0.29)	0.00 (–0.37, 0.36)

[1]Exposures have been log2–transformed and models have been adjusted for all listed exposures, child race, sex, age at exam, year of birth, and HOME score; maternal marital status at child's birth, IQ, seafood consumption during pregnancy, and smoking during pregnancy; maternal and paternal education and annual household income at child's birth; study examiner. [2]Secondary analysis group: complete inhibition outcome, covariate and exposure data for DDE, HCB, ΣPCB_4, Pb, Mn, MeHg, As, total *n*=235. Abbreviations: DDE: dichlorodiphenyldichloroethylene; HCB: hexachlorobenzene; ΣPCB_4: Sum of 4 PCB congeners (118, 138, 153, 180); Pb: lead; Mn: manganese; MeHg: methylmercury; As: arsenic.

Table S8. Inverse probability weighted results of multivariable linear regression analyses (difference in scaled scores associated with a twofold increase in exposure and 95% CI)[1] assessing the relation of prenatal exposure to a seven–chemical mixture with Delis Kaplan Executive Function System inhibition scaled scores among adolescents in the secondary analysis group[2].

**Exposure**	**Design Fluency total correct scaled score Difference (95% CI)**	**Color–Word Interference completion time scaled score Difference (95% CI)**
Log_2 DDE	0.04 (–0.37, 0.45)	0.27 (–0.16, 0.70)
Log_2 HCB	0.20 (–0.19, 0.59)	0.06 (–0.35, 0.47)
Log_2 ΣPCB_4	–0.25 (–0.72, 0.23)	–0.57 (–1.07, –0.08) *
Log_2 Pb	–0.12 (–0.52, 0.28)	0.12 (–0.31, 0.54)
Log_2 Mn	–0.27 (–1.01, 0.47)	–0.49 (–1.26, 0.29)
Log_2 MeHg	–0.29 (–0.67, 0.09)	0.37 (–0.02, 0.77)
Log_2 As	–0.05 (–0.42, 0.32)	0.00 (–0.39, 0.39)

[1]Exposures have been log2–transformed and models have been adjusted for all listed exposures, child race, sex, age at exam, year of birth, and HOME score; maternal marital status at child's birth, IQ, seafood consumption during pregnancy, and smoking during pregnancy; maternal and paternal education and annual household income at child's birth; study examiner. [2]Secondary analysis group: complete inhibition outcome, covariate and exposure data for DDE, HCB, ΣPCB_4 Pb, Mn, MeHg, and As, n = 235. Abbreviations: DDE: dichlorodiphenyldichloroethylene; HCB: hexachlorobenzene; ΣPCB_4: Sum of 4 PCB congeners (118, 138, 153, 180); Pb: lead; Mn: manganese; MeHg: methylmercury; As: arsenic.
